# Inclusion bodies of human parainfluenza virus type 3 inhibit antiviral stress granule formation by shielding viral RNAs

**DOI:** 10.1371/journal.ppat.1006948

**Published:** 2018-03-08

**Authors:** Zhulong Hu, Yuang Wang, Qiaopeng Tang, Xiaodan Yang, Yali Qin, Mingzhou Chen

**Affiliations:** State Key Laboratory of Virology and Modern Virology Research Center, College of Life Sciences, Wuhan University, LuoJia Hill, Wuhan, China; Vanderbilt University Medical Center, UNITED STATES

## Abstract

Viral invasion triggers the activation of the host antiviral response. Besides the innate immune response, stress granules (SGs) also act as an additional defense response to combat viral replication. However, many viruses have evolved various strategies to suppress SG formation to facilitate their own replication. Here, we show that viral mRNAs derived from human parainfluenza virus type 3 (HPIV3) infection induce SG formation in an eIF2α phosphorylation- and PKR-dependent manner in which viral mRNAs are sequestered and viral replication is inhibited independent of the interferon signaling pathway. Furthermore, we found that inclusion body (IB) formation by the interaction of the nucleoprotein (N) and phosphoprotein (P) of HPIV3 correlated with SG suppression. In addition, co-expression of P with N_L478A_ (a point mutant of N, which is unable to form IBs with P) or with NΔN10 (lacking N-terminal 10 amino acids of N, which could form IBs with P but was unable to synthesize or shield viral RNAs) failed to inhibit SG formation, suggesting that inhibition of SG formation also correlates with the capacity of IBs to synthesize and shield viral RNAs. Therefore, we provide a model whereby viral IBs escape the antiviral effect of SGs by concealing their own newly synthesized viral RNAs and offer new insights into the emerging role of IBs in viral replication.

## Author summary

Human parainfluenza virus type 3 (HPIV3) is one of the major causes of acute respiratory tract diseases such as pneumonia and bronchitis in infants and children. Virus invasion activates cellular stress responses. One of these responses is the formation of SGs which counteract viral replication. However, many viruses have evolved various strategies to suppress SG formation, thus facilitating their own replication. We sought to determine if (and how) HPIV3 modulates SG formation to facilitate its replication and found that the viral messenger RNAs (mRNAs) of HPIV3 trigger SG formation in infected cells. As time increased post-infection, the number of cells containing SGs increased as well. To escape this response, HPIV3 forms IBs that shield viral RNAs, thereby preventing SG formation and allowing the virus to replicate and survive—and potentially invade other cells.

## Introduction

Human parainfluenza virus type 3 (HPIV3), a member of the *Paramyxoviridae* family, can cause acute respiratory tract diseases such as pneumonia and bronchitis in infants and children [1]. The genome of HPIV3 contains a non-segmented negative-strand RNA, which encodes 6 proteins: the nucleoprotein (N), phosphoprotein (P), matrix protein (M), fusion protein (F), hemagglutinin/neuraminidase (HN), and large RNA-dependent RNA polymerase (L) [2]. The virus enters host cells through a membrane fusion process carried out by HN and F [3]. P bridges L to the N-encapsidated genomic RNA to initiate viral transcription and replication [4–6], which generates 6 capped and polyadenylated messenger RNAs (polyA^+^ mRNAs) and a full-length anti-genomic RNA [7]. The new synthesized viral proteins and genomic RNA assemble into virions, which are released from the cell through the budding process [8,9].

Viral invasion triggers host antiviral defense responses, including the well-known innate immune response and the protein kinase R (PKR)-dependent stress response [10,11]. On one hand, viral RNAs synthesized during the infection are usually regarded as non-self, pathogen-associated molecular patterns (PAMPs). The PAMPs can be detected by retinoic acid-inducible gene I (RIG-I) and melanoma differentiation-associated protein 5 (MDA5) [12–16], and this detection activates the interferon (IFN) signaling pathway and results in the expression of IFN-α/β. Secreted IFN-α/β induces the transcription and expression of IFN-stimulated genes (ISGs), which play a critical role in restricting viral replication and propagation in host cells [17–19]. On the other hand, particular RNA species such as double-stranded RNA (dsRNA) or secondary-structured RNA generated during viral infection can also be detected by PKR [20,21]. This recognition activates PKR auto-phosphorylation, which results in the phosphorylation of eukaryotic initiation factor 2 α subunit (eIF2α). Phosphorylated eIF2α prevents the assembly of the ternary pre-initiation complex and inactivates global protein synthesis. Stalled translation initiation complexes are then recruited into the cytoplasmic aggregates, which are termed SGs [22]. Thus, SGs typically consist of stalled mRNAs, 40S ribosomal subunits, various translation initiation factors such as eIF3, eIF4A, eIF4E, and eIF4G, and several SG-nucleating factors, including G3BP [23–25]. As viral protein synthesis must rely on the host translation machinery, SG-mediated translational arrest usually plays an antagonistic role in the viral life cycle [26].

Given the inhibitory effect of SGs on viral infection, many viruses have evolved various strategies to block SG formation to ensure efficient viral replication. Sendai virus (SeV) and measles virus (MV) encode a C protein to limit the accumulation of dsRNA to inhibit SG formation [27,28]. Influenza A virus (IAV) NS1 protein prevents viral dsRNA from being detected by PKR, thus resulting in the inhibition of SG formation [29,30]. Poliovirus infection induces SG formation at the early stage of viral infection; then, SGs gradually disappear due to the cleavage of G3BP by viral 3C protease [31]. Respiratory syncytial virus (RSV) sequesters p38 and OGT into viral IBs to suppress SG assembly [32]. Ebola virus (EBOV) IBs sequester various SG markers and inhibit SG formation during infection [33,34].

In this report, we show that viral mRNAs derived from HPIV3 infection induce eIF2α phosphorylation and trigger SG formation in a PKR-dependent manner. HPIV3-induced SGs play an inhibitory role in HPIV3 replication by sequestering viral mRNAs. Disruption of SG formation by different approaches dramatically increases viral protein expression and virion production without influencing IFN production. Furthermore, we show that HPIV3 IBs formed during infection block SG formation by shielding viral RNAs.

## Results

### HPIV3 infection induces SG formation

**Fig 1:**
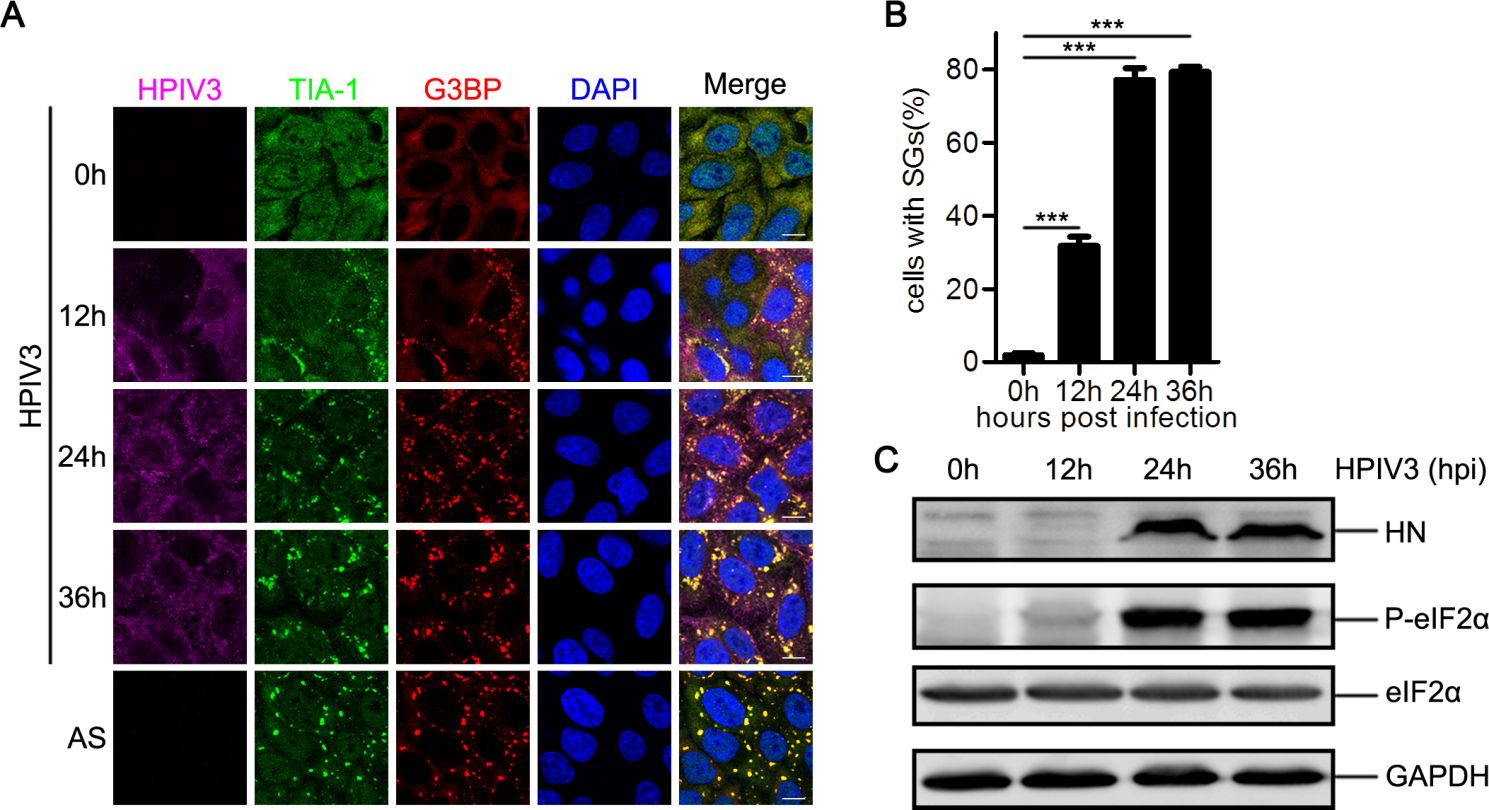
HPIV3 infection induces SG formation. (A, B, and C) HeLa cells were mock-treated, treated with AS (0.5 mM) for 1 h, or infected with HPIV3 (MOI = 1). At the indicated time points pi, (A) cells were analyzed by confocal microscopy after being immunostained for HPIV3 (purple), TIA-1 (green), and G3BP (red). Nuclei were stained with DAPI (blue). The white scale bar corresponds to 10 μm. (B) The percentage of cells containing SGs was quantified in three independent experiments. At least 100 cells were counted each time. Data are represented as means ±SD. Student’s t test: * P<0.05, ** P<0.01, *** P<0.001, ns = not significant. (C) Cell lysates were analyzed via western blot using anti-HN, anti-phosphorylated eIF2α, anti-eIF2α, and anti-GAPDH antibodies.

To determine whether HPIV3 infection induces SG formation, we infected HeLa cells with HPIV3 for up to 36 hour (h) and analyzed the distribution of SG marker proteins TIA-1 and G3BP at different time points post-infection (pi). An antibody against HPIV3 was used to evaluate viral protein expression and viral replication (Fig 1A). In mock-infected cells, TIA-1 was homogeneously distributed in both the cytoplasm and the nuclei and G3BP was homogeneously distributed in the cytoplasm. In contrast, TIA-1 translocated from the nuclei to the cytoplasm and formed aggregates co-localizing with G3BP during viral infection, which was similar to the results after sodium arsenite (AS) stimulation (Fig 1A). As infection progressed, the number of cells containing SGs increased to nearly 80% at 24 hpi and this ratio remained stable until 36 hpi (Fig 1B). To determine whether HPIV3-induced SGs contain other typical SG marker proteins, we also examined the distribution of eIF4A, eIF4E, and eIF4G and found that all these marker proteins were re-distributed into SGs and co-localized with G3BP during HPIV3 infection (S1A Fig). To determine whether HPIV3-induced SGs are cell-specific, we also examined the distribution of TIA-1 and G3BP in HEp-2 cells and MK2 cells infected with HPIV3 and found that both TIA-1 and G3BP were re-distributed into SGs and co-localized with each other, suggesting that SG formation is a general process during HPIV3 infection (S1B Fig).

Since the phosphorylation of eIF2α is critical for SG formation, we evaluated the phosphorylation status of eIF2α in HPIV3-infected cells and found that the level of phosphorylated eIF2α increased at 12 hpi, peaked at 24 hpi, and remained stable until 36 hpi. (Fig 1C). Furthermore, we examined the distribution of phosphorylated eIF2α via immunofluorescence and found that little phosphorylated eIF2α was detected in mock-treated cells. In contrast, both HPIV3 infection and AS stimulation obviously increased the level of phosphorylated eIF2α, and phosphorylated eIF2α was recruited into SGs, which co-localized with G3BP (S1C Fig), suggesting that HPIV3 infection activates eIF2α phosphorylation and subsequently induces SG formation.

**Fig 2:**
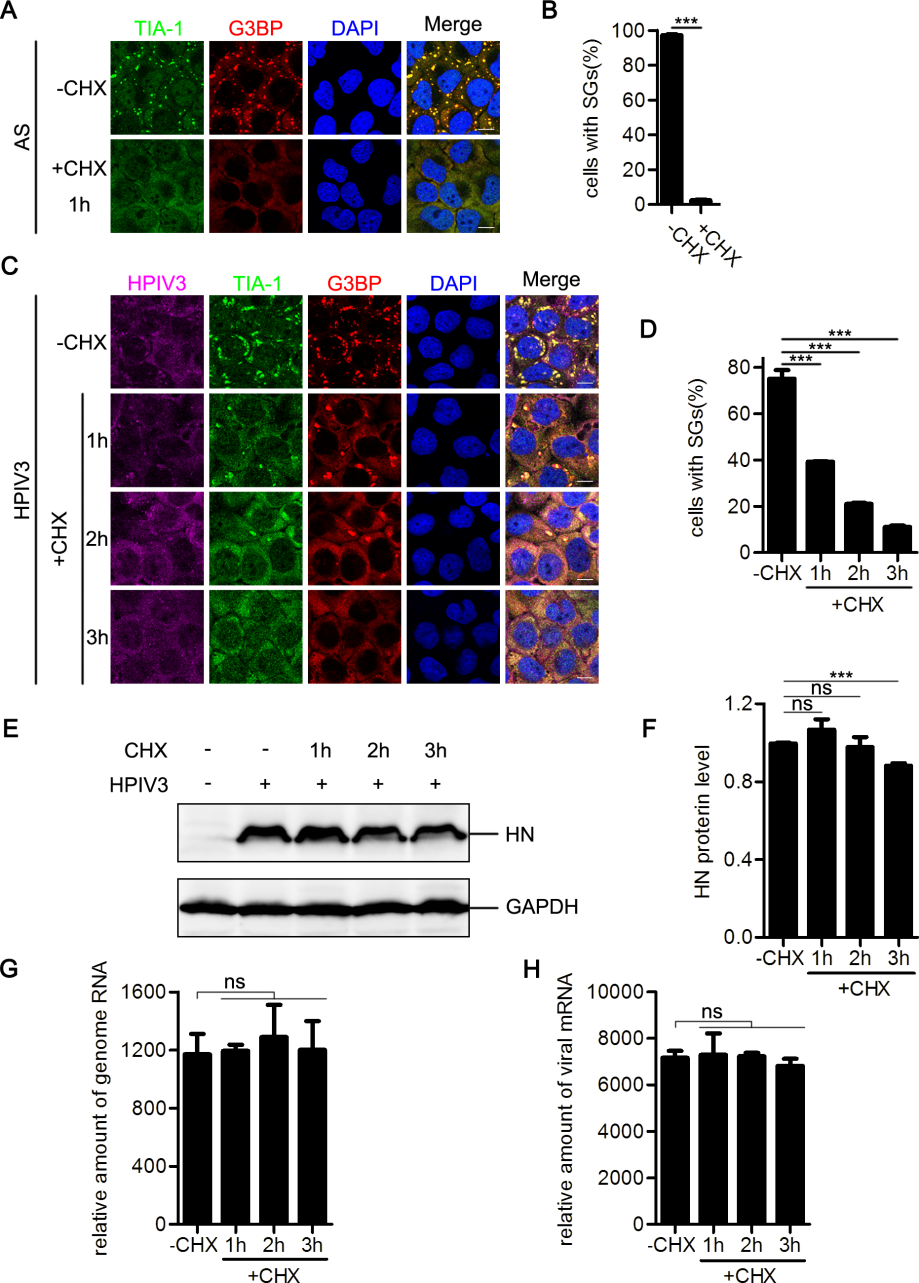
CHX treatment induces HPIV3-triggered SG disassembly. (A and B) HeLa cells were treated with AS (0.5 mM) for 30 min and subsequently treated with or without CHX in the presence of AS for another 1 h. (A) Cells were immunostained for TIA-1 (green) and G3BP (red). Nuclei were stained with DAPI (blue). The white scale bar corresponds to 10 μm. (B) The percentage of cells containing SGs was quantified in three independent experiments. (C-H) HeLa cells were mock-infected or infected with HPIV3 (MOI = 1) for 24 h and subsequently mock-treated or treated with CHX for another 1 h, 2 h, or 3 h. (C) Cells were immnostained for HPIV3 (purple), TIA-1 (green), and G3BP (red). Nuclei were stained with DAPI (blue). The white scale bar corresponds to 10 μm. (D) The percentage of cells containing SGs was quantified in three independent experiments. (E) Cell lysates were analyzed via western blot using anti-HN and anti-GAPDH antibodies. (F) Western blots from three independent experiments were quantified and normalized with respect to the amount of GAPDH. (G and H) Total RNA were isolated for qPCR analysis to measure the indicated RNA abundance and normalized to that of GAPDH. Data are represented as means ±SD. Student’s t test: * P<0.05, ** P<0.01, *** P<0.001, ns = not significant.

Assembly of SGs is a highly dynamic process, and phosphorylation of eIF2α inhibits translational initiation, allowing mRNA transcripts release from the elongating ribosomes and accumulate at SGs under environmental stress. Drugs that stabilize polysomes inhibit the assembly of SGs, whereas drugs that destabilize polysomes promote the assembly of SGs. cycloheximide (CHX) stabilize polysomes by freezing ribosomes on translating mRNAs and inhibit SG formation[35]. Previous studies reported that some viruses trigger the formation of atypical SGs that do not disassemble in the presence of CHX [36,37]. We next sought to determine whether HPIV3 infection- triggered SGs disassemble in response to CHX. CHX was added to cells in the presence of AS or HPIV3 infection. SGs induced by AS were cleared via incubation with CHX for 1 h (Fig 2A and 2B). Similarly, after incubation with CHX for 3 h, HPIV3-induced SGs completely disappeared (Fig 2C and 2D). CHX treatment results in global inhibition of protein synthesis. We examined the viral HN protein level via western blotting and found that HN protein expression was inhibited by incubation with CHX (Fig 2E and 2F). To rule out possible effect of CHX on viral RNA synthesis, we also examined the viral RNA level after treatment with CHX, and found that neither viral genomic RNA nor mRNA synthesis was significantly affected in the presence of CHX (Fig 2G and 2H). These data suggest that HPIV3 infection induces the formation of canonical SGs.

### mRNAs of HPIV3 trigger SG formation

**Fig 3:**
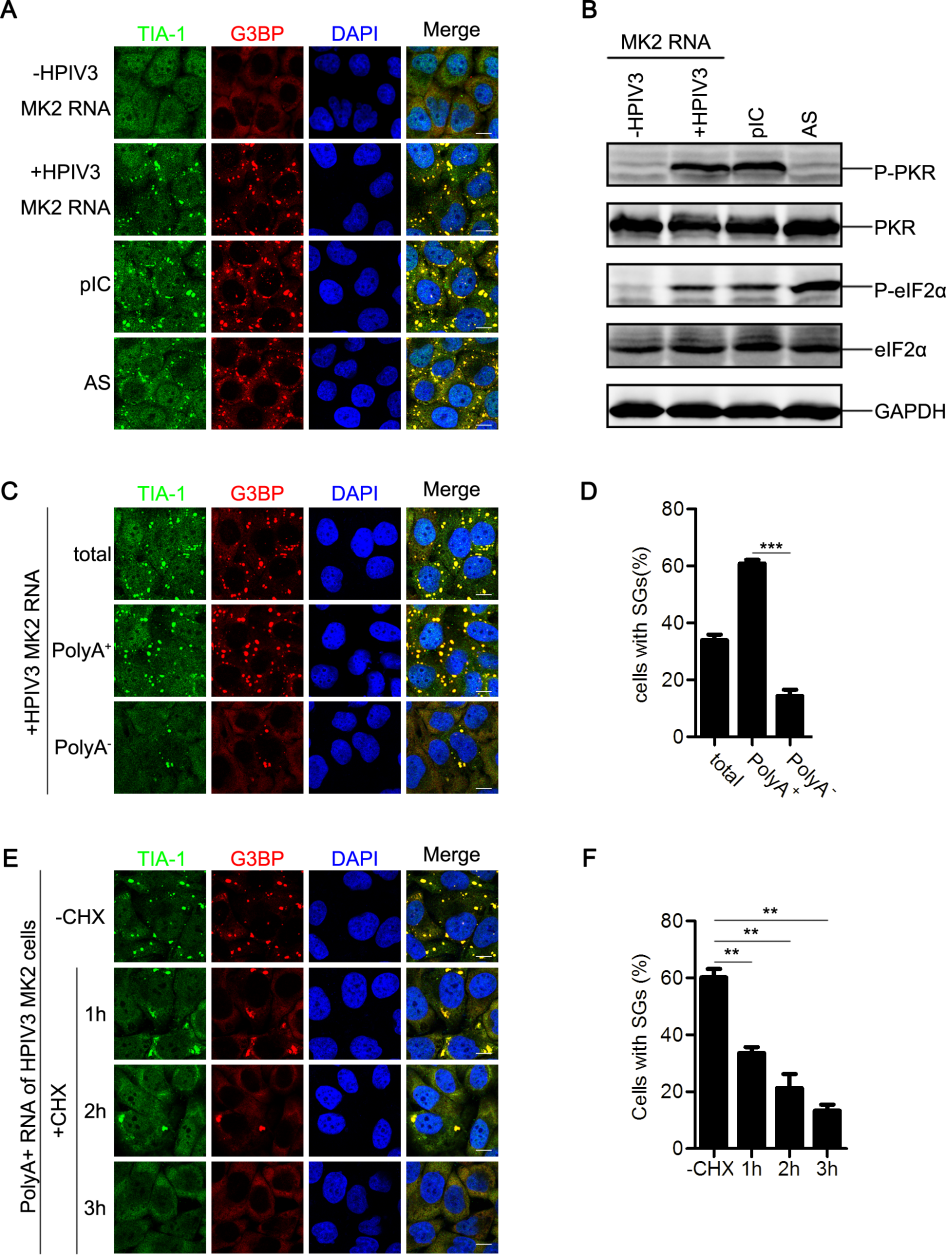
HPIV3 viral RNA triggers SG formation. (A and B) HeLa cells were transfected with the indicated RNA samples from mock-infected or HPIV3-infected MK2 cells, treated with pIC for 12 h or AS for 1 h. (A) Cells were immunostained for TIA-1 (green) and G3BP (red). Nuclei were stained with DAPI (blue). The white scale bar corresponds to 10 μm. (B) Cell lysates were analyzed via western blot using anti-phosphorylated PKR, anti-PKR, anti-phosphorylated eIF2α, anti-eIF2α, and anti-GAPDH antibodies. (C and D) HeLa cells were transfected with the RNA from HPIV3-infected MK2 cells, the respective PolyA^+^ RNA fraction, or the respective PolyA^-^ RNA fraction for 12 h. (C) Cells were immunostained for TIA-1 (green) and G3BP (red). Nuclei were stained with DAPI (blue). The white scale bar corresponds to 10 μm. (D) The percentage of cells containing SGs was quantified in three independent experiments. (E and F) HeLa cells were transfected with the respective PolyA^+^ RNA fraction from HPIV3-infected MK2 cells for 12 h and subsequently mock-treated or treated with CHX for another 1 h, 2 h, or 3 h. (E) Cells were immunostained for TIA-1 (green) and G3BP (red). Nuclei were stained with DAPI (blue). The white scale bar corresponds to 10 μm. (F) The percentage of cells containing SGs was quantified in three independent experiments. Data are represented as means ±SD. Student’s t test: * P<0.05, ** P<0.01, *** P<0.001, ns = not significant.

HPIV3 infection induces SG formation, suggesting that either the viral RNAs or the viral proteins or both of them induce SG formation. N, P, M, F, and HN were separately expressed, but G3BP was always homogeneously distributed in the cytoplasm (S2A Fig), suggesting that none of them triggered SG formation. Furthermore, over-expression of HPIV3 viral proteins could not induce the phosphorylation of eIF2α either (S2B Fig), suggesting that SGs triggered by HPIV3 infection is not due to the accumulation of viral proteins. Then, we speculated that viral RNAs generated from viral replication and transcription might be the critical factors for inducing SG formation in host cells. We extracted RNAs from mock- or HPIV3-infected cells. These RNAs were subsequently transfected into HeLa cells that were immunostained with antibodies against TIA1 and G3BP to detect SG formation. We found that RNAs extracted from mock-infected MK2 cells failed to induce TIA-1 and G3BP aggregation into SGs. In contrast, RNAs extracted from HPIV3-infected MK2 cells induced the formation of a large number of SGs (Fig 3A, compare panel “-HPIV3 MK2 RNA” to “+HPIV3 MK2 RNA”). As a positive control, Poly I:C (pIC) also stimulated SG formation (Fig 3A), suggesting that RNAs derived from HPIV3-infected cells trigger SG formation. The time course experiment showed that small SGs appeared at 4 h post-transfection. As time extended, the SGs gradually became larger and reached the maximum at 12 h post-transfection (S2C and S2D Fig).

Several RNA viruses have been reported to activate eIF2α phosphorylation through activation of the upstream kinase PKR [20]. Having found that HPIV3 infection activated eIF2α phosphorylation, we sought to determine whether RNAs derived from HPIV3-infected cells could activate PKR/eIF2α phosphorylation. pIC transfection induced PKR and eIF2α phosphorylation (Fig 3B). Similarly, RNAs extracted from HPIV3-infected cells also activated PKR/eIF2α phosphorylation. In contrast, PKR/eIF2α phosphorylation was not detected using RNAs extracted from mock-infected cells (Fig 3B). As expected, AS treatment failed to activate PKR phosphorylation, but significantly activated eIF2α phosphorylation (Fig 3B), which is consistent with previous research results showing that AS induces SG formation in a PKR-independent manner [38,39]. Taken together, these results show that HPIV3 viral RNAs activate PKR/eIF2α phosphorylation, thus inducing SG formation.

Because viral replication and transcription generate genome RNAs, anti-genome RNAs, mRNAs and dsRNA, we sought to determine which RNAs play a critical role in inducing SG formation. For this purpose, we isolated PolyA^+^ RNAs (viral mRNAs) from RNAs derived from HPIV3-infected cells, and the remaining fraction was termed as PolyA^-^ RNAs. Both fractions were evaluated for the induction of SG formation. PolyA^+^ RNAs had a much greater ability to induce SG formation than PolyA^-^ RNAs (Fig 3C and 3D). Furthermore, we also found that *in vitro* transcribed HPIV3 N mRNA could also induce SG formation (S2E Fig). Therefore, we concluded that mRNAs of HPIV3 trigger SG formation.

Next, we sought to know whether the SGs induced by transfection of PolyA^+^ RNAs shares the same property with the SGs induced by HPIV3 infection. CHX were added to cells transfected with PolyA^+^ RNAs derived from HPIV3-infected cells, and results showed that SGs induced by PolyA^+^ RNAs from HPIV3-infected cells also gradually disassembled in the presence of CHX (Fig 3E and 3F), which is in accordance with the result of HPIV3 infection.

### HPIV3 triggers SG formation in a PKR-dependent manner

**Fig 4:**
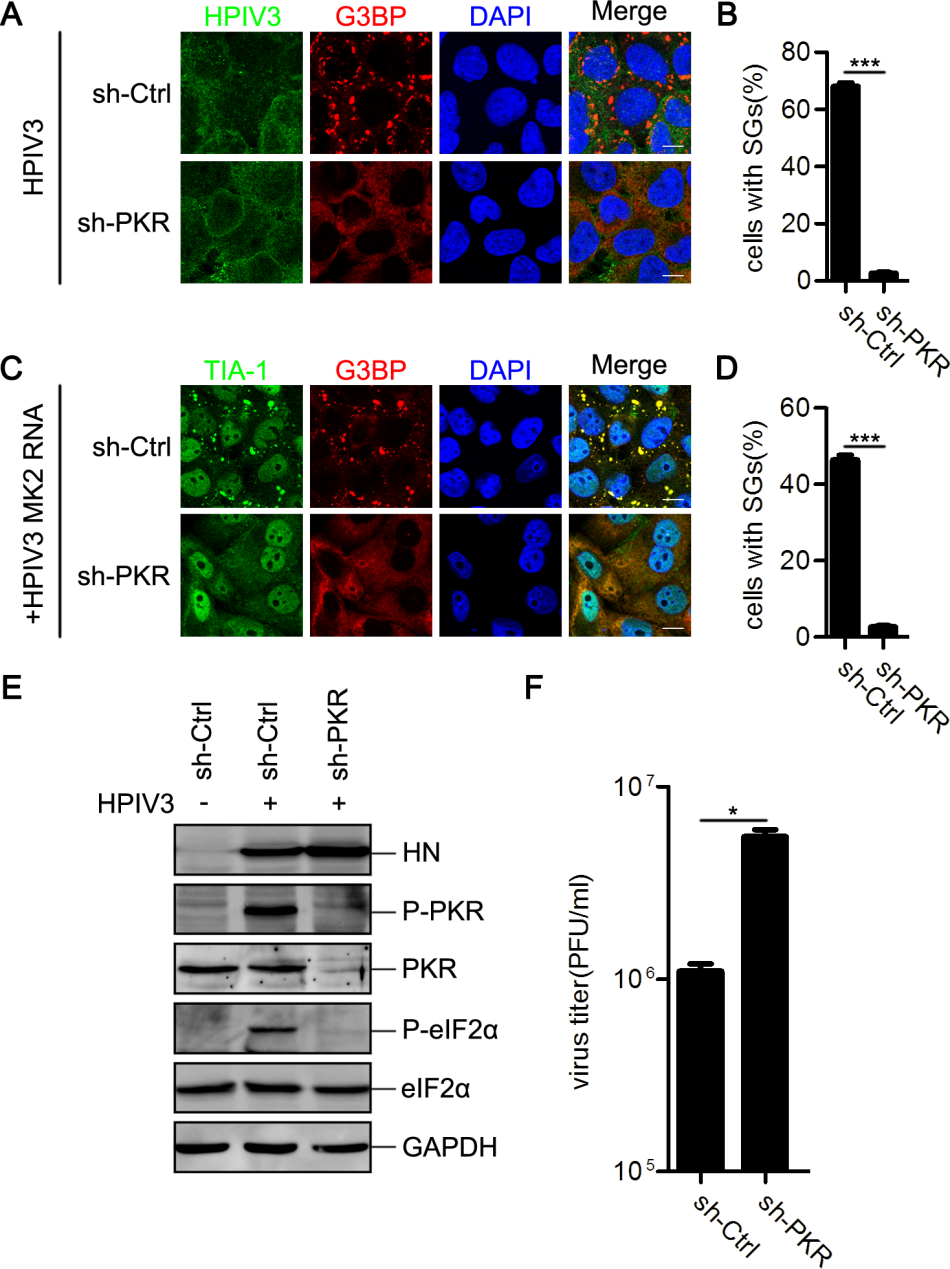
HPIV3 triggers SG formation in a PKR-dependent manner. (A and B) HeLa cells with or without PKR knockdown were infected with HPIV3 (MOI = 1) for 24 h. (A) Cells were immunostained for HPIV3 (green) and G3BP (red). Nuclei were stained with DAPI (blue). The white scale bar corresponds to 10 μm. (B) The percentage of cells containing SGs was quantified in three independent experiments. (C and D) HeLa cells with or without PKR knockdown were transfected with RNA from HPIV3-infected MK2 cells for 12 h. (C) Cells were immunostained for TIA-1 (green) and G3BP (red). Nuclei were stained with DAPI (blue). The white scale bar corresponds to 10 μm. (D) The percentage of cells containing SGs was quantified in three independent experiments. (E and F) HeLa cells with or without PKR knockdown were mock-infected or infected with HPIV3 (MOI = 1) for 24 h. (E) Cell lysates were analyzed via western blot using anti-HN, anti-phosphorylated PKR, anti-PKR, anti-phosphorylated eIF2α, anti-eIF2α, and anti-GAPDH antibodies. (F) The supernatants were collected for a plaque assay to determine the viral titer. Data are represented as means ±SD. Student’s t test: * P<0.05, ** P<0.01, *** P<0.001, ns = not significant.

We next sought to confirm whether HPIV3 infection induces SG formation in a PKR-dependent manner. We created cell lines to induce stable knockdown of PKR expression by transducing HeLa cells with lentiviral shRNA transduction vector. HPIV3 infection obviously induced SG formation in mock-knockdown cells (Fig 4A, panel “sh-ctrl,” and 4B). In contrast, PKR-knockdown cells failed to form SGs upon HPIV3 infection (Fig 4A, panel “sh-PKR,” and 4B). Similarly, neither RNAs from HPIV3-infected cells nor pIC induced SG formation in PKR-knockdown cells (Fig 4C and 4D; S3A and S3B Fig), suggesting that HPIV3 induced SG formation in a PKR-dependent manner. Because SGs were still able to form upon AS treatment in PKR-knockdown cells (S3C and S3D Fig), the inhibition of SG formation in PKR-knockdown cells during HPIV3 infection or pIC treatment was not due to an intrinsic defect of cells in response to stress.

Next, we detected the phosphorylation level of PKR and eIF2α in both mock- and PKR-knockdown cells during HPIV3 infection. In mock-knockdown cells, HPIV3 infection obviously induced PKR/eIF2α phosphorylation, whereas in PKR-knockdown cells, phosphorylated PKR/eIF2α was not detected (Fig 4E), suggesting that HPIV3-induced eIF2α phosphorylation depends on PKR phosphorylation. Interestingly, knockdown of PKR expression significantly increased viral HN protein expression and viral titers (Fig 4E and 4F), indicating that HPIV3-induced SGs may play an inhibitory role in viral replication.

### Inhibition of SG formation facilitates HPIV3 replication

**Fig 5:**
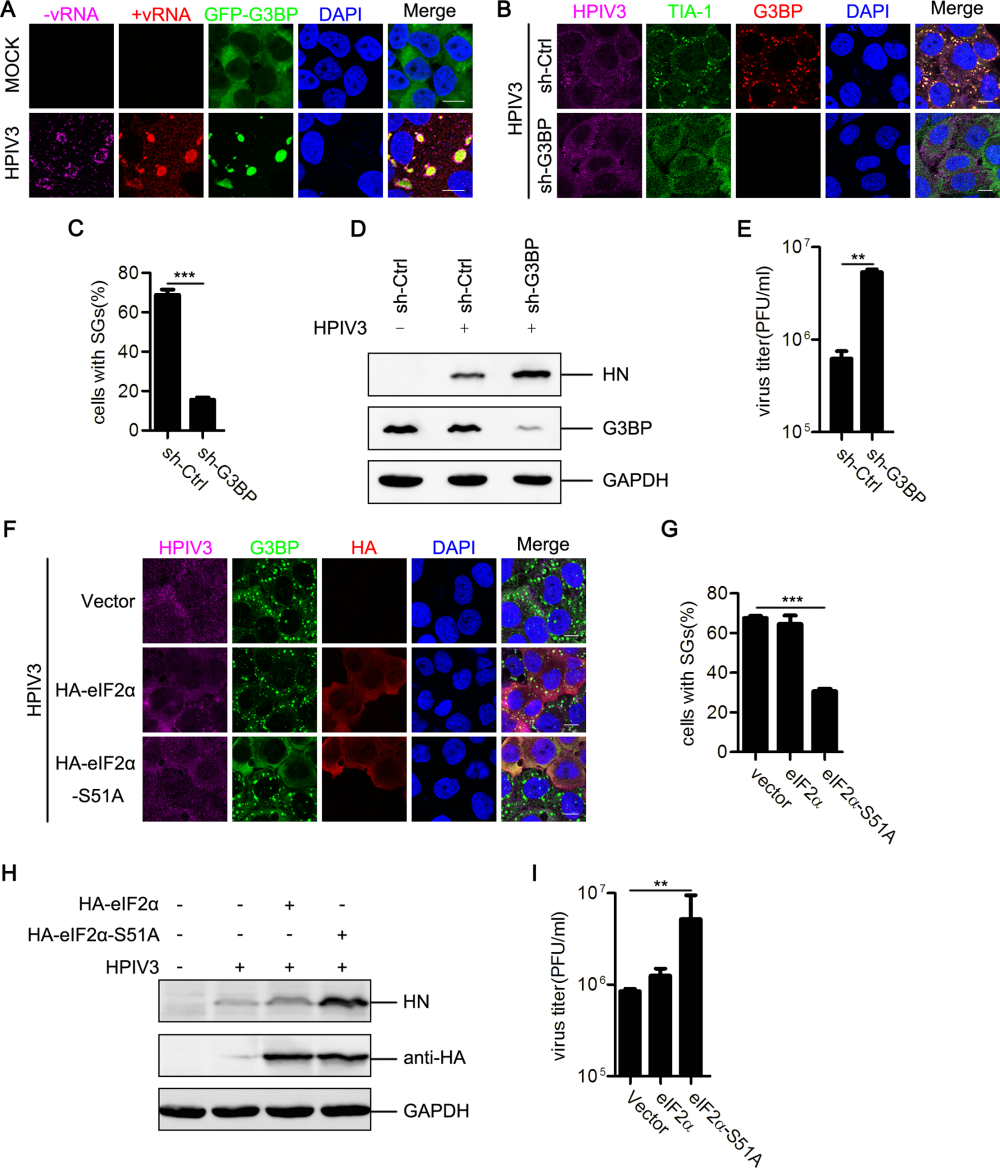
Inhibition of SG formation facilitates HPIV3 replication. (A) HeLa-GFP-G3BP cells were mock-infected or infected with HPIV3 (MOI = 1) for 24 h. HPIV3 -vRNA (purple) and +vRNA (red) were detected via RNA-FISH. Nuclei were stained with DAPI (blue). The white scale bar corresponds to 10 μm. (B-E) Wild type or G3BP-deficient HeLa cells were infected with HPIV3 (MOI = 1) for 24 h. (B) Cells were immunostained for HPIV3 (purple), TIA-1 (green), and G3BP (red). Nuclei were stained with DAPI (blue). The white scale bar corresponds to 10 μm. (C) The percentage of cells containing SGs was quantified in three independent experiments. (D) Cell lysates were analyzed via western blot using anti-HN, anti-G3BP, and anti-GAPDH antibodies. (E) The supernatants were collected for a plaque assay to determine the viral titer. (F-I) HeLa cells were transfected with an empty plasmid or plasmids encoding eIF2α or the nonphosphorylatable mutant, eIF2α-S51A, for 24 h, then infected with HPIV3 (MOI = 1) for additional 24 h. (F) Cells were immunostained for HPIV3 (purple), G3BP (green), and HA tag (red). Nuclei were stained with DAPI (blue). The white scale bar corresponds to 10 μm. (G) The percentage of cells containing SGs was quantified in three independent experiments. (H) Cell lysates were analyzed via western blot using anti-HN, anti-HA, and anti-GAPDH antibodies. (I) The supernatants were collected for a plaque assay to determine the viral titer. Data are represented as means ±SD. Student’s t test: * P<0.05, ** P<0.01, *** P<0.001, ns = not significant.

Several viruses have been reported to trigger the formation of SGs that capture viral RNAs and suppress viral replication [26]. We next sought to evaluate the distribution of viral RNAs in HPIV3-infected cells. A cell line stably expressing GFP-G3BP was used to perform the RNA fluorescent in situ hybridization (RNA-FISH) assay. GFP-G3BP in HPIV3-infected cells was recruited into SGs (Fig 5A). Viral positive-strand RNAs (+vRNAs, including the mRNA, the most abundant viral RNA and the antigenome RNA) and genomic RNAs (-vRNAs) were detected with specific probes in HPIV3-infected cells (Fig 5A). Clearly, +vRNAs co-localized well with SGs, but -vRNAs were enriched adjacent to and encircle the SGs (Fig 5A), suggesting that HPIV3-induced SGs can capture +vRNAs. Since viral mRNAs are the major component of +vRNAs, we hypothesized that SGs sequester viral mRNAs to suppress viral protein translation, thereby inhibiting viral replication.

Having established that knockdown of PKR expression significantly increases HN expression and HPIV3 titers, and HPIV3-induced SGs capture viral mRNAs, we sought to determine whether SG formation inhibits HPIV3 replication. Because G3BP is a core factor in SG formation, we first knocked down G3BP expression by shRNA and found that minimal G3BP could be observed in G3BP-knockdown cells, while TIA-1 expression was substantial (Fig 5B). The percentage of G3BP-knockdown cells containing SGs decreased markedly upon HPIV3 infection (Fig 5B and 5C) or AS treatment (S3E and S3F Fig), suggesting that knockdown of G3BP expression significantly impairs SG formation. We then detected HN expression and HPIV3 titers and found that inhibition of SG formation significantly enhanced HN expression (Fig 5D) and viral production (Fig 5E), indicating that SGs play an antiviral role. To exclude the possibility that the antiviral activity of G3BP is inherent, we over-expressed an HA-tagged non-phosphorylatable mutant of eIF2α, eIF2α-S51A (HA-eIF2α-S51A), in HeLa cells, and found that eIF2α-S51A expression resulted in deficient SG formation, but eIF2α expression had no effect on SG formation upon HPIV3 infection (Fig 5F and 5G) or AS stimulation (S3G and S3H Fig). Similarly, inhibition of SG formation caused by eIF2α-S51A expression significantly enhanced HN expression and viral production, but eIF2α expression had no effect on either HN expression or viral production (Fig 5H and 5I). Taken together, these data suggest that HPIV3-induced SG formation has an antiviral effect, and inhibition of SG formation enhances HPIV3 replication.

### IFN induction is independent on SG formation in HPIV3-infected cells

**Fig 6:**
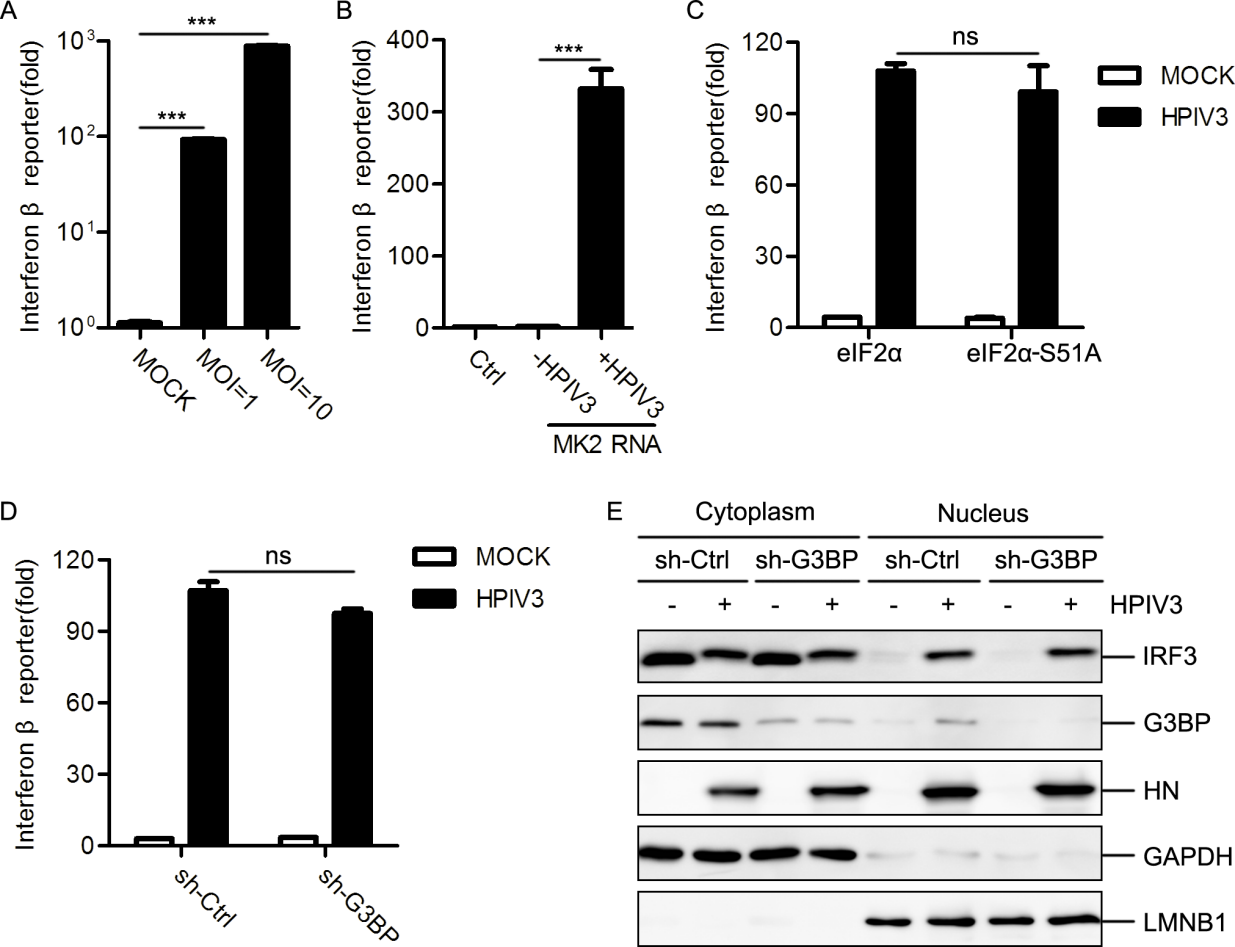
Relationship between SG formation and innate immune response. (A) HEK293T cells were transfected with 50 ng IFNβ-Luc reporter and 20 ng TK-Luc reporter for 12 h, then mock-infected or infected with HPIV3 (MOI = 1 or 10) for 24 h. Cells were harvested for a luciferase assay. (B) HEK293T cells were transfected with 50 ng IFNβ-Luc reporter and 20 ng TK-Luc reporter together with the indicated RNA samples for 24 h. Cells were harvested for a luciferase assay. (C) HEK293T cells were transfected with 50 ng IFNβ-Luc reporter and 20 ng TK-Luc reporter together with the indicated plasmid encoding eIF2α and eIF20078-S51A for 24 h, then mock-infected or infected with HPIV3 (MOI = 1) for another 24h. Cells were harvested for a luciferase assay. (D) HEK293T cells with or without G3BP knockdown were transfected with 50 ng IFNβ-Luc reporter and 20 ng TK-Luc reporter for 12 h, then mock-infected or infected with HPIV3 (MOI = 1) for 24 h. Cells were harvested for a luciferase assay. (E) HEK293T cells with or without G3BP knockdown were mock-infected or infected with HPIV3 (MOI = 1) for 24 h. Cells were harvested to extract the nuclei fraction and the cytosol fraction. Protein samples were analyzed via western blot using anti-IRF3, anti-G3BP, anti-HN, anti-GAPDH, and anti-LMNB1 antibodies. Data are represented as means±SD. Student’s t test: * P<0.05, ** P<0.01, *** P<0.001, ns = not significant.

HPIV3 infection generates various viral RNAs in which dsRNA, the 5’-triphosphate of genome and the anti-genome RNA are generally considered as an inducer of IFN production [13,40]. We confirmed that HPIV3 infection and RNAs extracted from HPIV3-infected cells indeed efficiently induced IFN expression (Fig 6A and 6B), suggesting that viral RNAs derived from HPIV3 infection can not only induce SG formation but also activate IFN production. Next, we sought to know whether there is a link between IFN induction and SG formation. First, we over-expressed the caspase activation and recruitment domain (CARD) of RIG-I (RIG-I-N) or VISA (also known as MAVS, IPS-1 or cardif) to activate the RLR pathway, and found that over-expression of RIG-I-N or VISA induced potently IFN expression, but failed to induce SG formation (S4A and S4B Fig). Furthermore, to rule out the possibility that activation of RLR pathway is required for SG formation, MEF cells-knocked out RIG-I or VISA were infected with HPIV3. The results showed that HPIV3 infection induced SG formation in MEF cells-knocked out RIG-I or VISA as efficiently as in wild-type MEF cells (S4C–S4E Fig). Taken together, these data suggest that IFN induction is not required for SG formation which is consistent with previous results that activation of the RLR pathway does not induce and is not required for SG formation[38]. Several studies have suggested that SGs may serve as a platform for IFN production [41–43], while others have pointed out that SGs are dispensable for the induction of innate immune response [38,44]. To determine whether HPIV3-induced SGs are involved in IFN response, we measured IFN response when SG formation was disrupted. We found that HPIV3 infection still potently induced IFN expression in spite of the inhibition of SG formation by over-expression of eIF2α-S51A (Fig 6C) or knockdown of G3BP expression (Fig 6D). Furthermore, HPIV3 infection equally induced IRF3 translocation into the nucleus in both mock-knockdown and G3BP-knockdown cells (Fig 6E), suggesting that SG formation is dispensable for the activation of IFN response.

### HPIV3 IBs inhibit SG formation

Since SG formation plays an antiviral role in HPIV3 infection, we next sought to determine whether viral proteins can inhibit SG formation to escape the antiviral response of SGs. First, we found that over-expression of M, F, or HN separately had no effect on SG formation in HPIV3-infected cells (S5A and S5B Fig).

**Fig 7:**
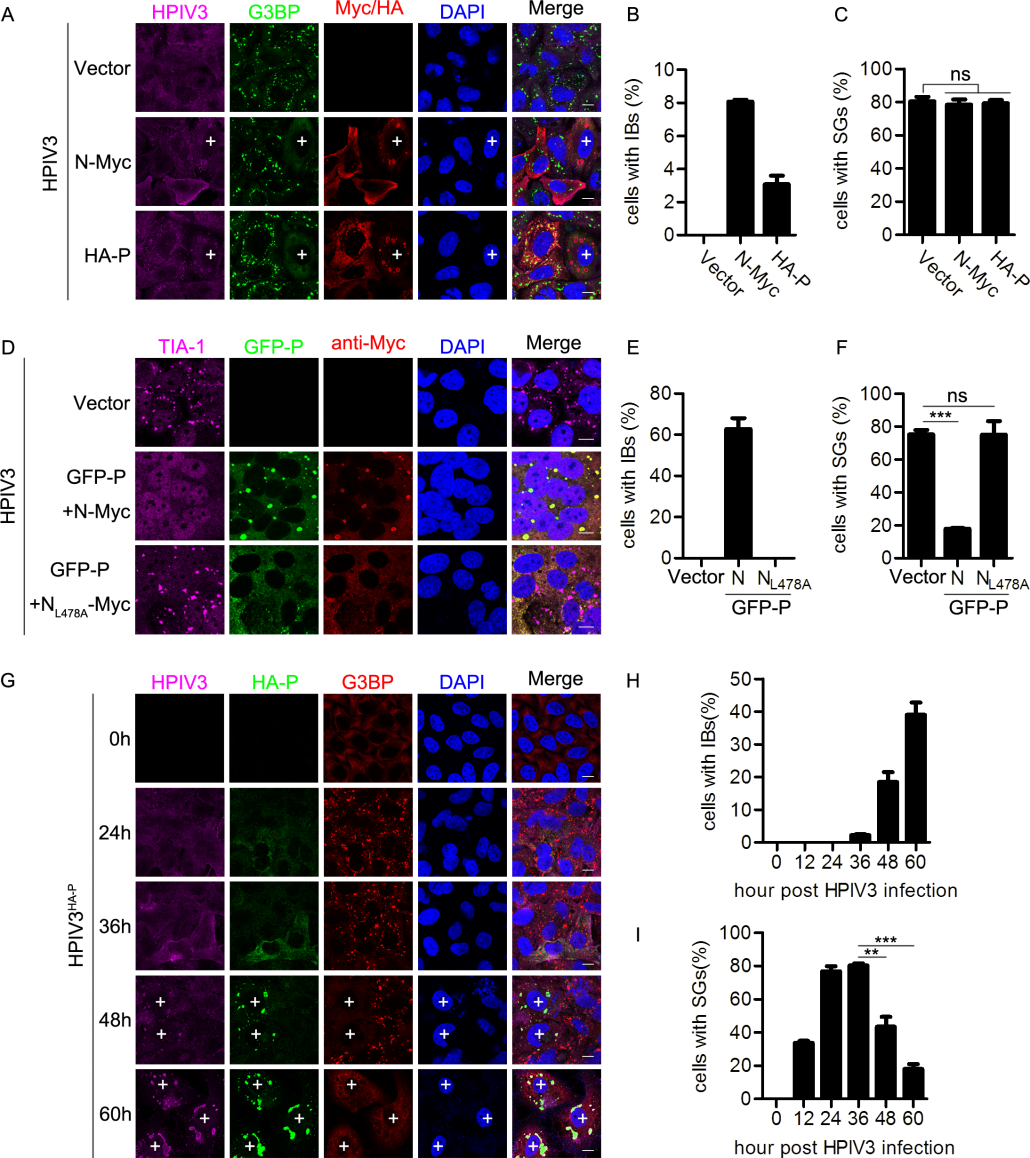
HPIV3 IBs inhibit HPIV3-induced SG formation. (A-C) HeLa cells were transfected with an empty plasmid or plasmid encoding N or P for 24 h, then infected with HPIV3 (MOI = 1) for another 24 h. (A) Cells were immunostained for HPIV3 (purple), G3BP (green), and Myc/HA tag (viral protein, red). Nuclei were stained with DAPI (blue). “+” indicates the cells containing IBs. The white scale bar corresponds to 10 μm. (B and C) The percentage of cells containing IBs or SGs was quantified in three independent experiments. (D-F) HeLa cells were transfected with an empty plasmid or co-transfected with plasmids encoding GFP-P and N-Myc or the mutant N_L478A-_Myc for 24h, then infected with HPIV3 (MOI = 1) for another 24 h. (D) Cells were immunostained for TIA-1 (purple) and Myc (red). Nuclei were stained with DAPI (blue). The white scale bar corresponds to 10μm. (E and F) The percentage of cells containing IBs or SGs was quantified in three independent experiments. (G-I) HeLa cells were either mock-infected or infected with HPIV3^HA-P^ (MOI = 10). At the indicated time points post-infection, (G) cells were immunostained for HPIV3 (purple), HA tag (green), and G3BP (red). Nuclei were stained with DAPI (blue). The white scale bar corresponds to 10 μm. (H and I) The percentage of cells containing IBs or SGs was quantified in three independent experiments. Data are represented as means ±SD. Student’s t test: * P<0.05, ** P<0.01, *** P<0.001, ns = not significant.

We also expressed N or P individually in HPIV3-infected cells (S5C Fig) and examined distribution of N or P (Fig 7A). We found that there was no significant difference for HPIV3-induced SG formation in the presence or absence of N or P, and over-expressed N or P distributed homogeneously in >90% of HPIV3-infected cells (Fig 7A–7C), but, to our surprise, we did find that ~8% of cells over-expressing N or ~3% of cells over-expressing P formed IBs in HPIV3-infected cells (due to over-expressed N interaction with P derived from HPIV3 infection or over-expressed P interaction with N derived from HPIV3 infection), and all the cells containing IBs appear to have no SGs (Fig 7A–7C; “+”implies a cell forming IBs), indicating that over-expression of N or P does not inhibit HPIV3-induced SG formation unless N or P form IBs, which also implies that IBs may play an inhibitory function on SG formation.

We previously reported that when co-expressed N and P of HPIV3 could form IBs, which were the center of HPIV3 viral RNA synthesis [7]. To confirm that IBs indeed inhibit SG formation, we over-expressed N and P of HPIV3 to force the formation ability of IBs in HPIV3-infected cells. We found that ~60% cells formed IBs, all the IBs-containing cells had no SGs and the percentage of cells containing SGs decreased from ~80% to ~20%. (Fig 7D, panel “GFP-P+N-Myc” and Fig 7E and 7F). However, co-expression of GFP-P with N_L478A_-Myc, a point mutant of N (we previously showed that N_L478A_ was unable to form IBs with P [7]), failed to inhibit SG formation in HPIV3-infected cells (Fig 7D, panel “GFP-P+N_L478A_-Myc” and Fig 7E and 7F), suggesting that the formation of IBs by over-expression of N and P indeed efficiently inhibit SG formation, and disruption of IB formation abolishes the inhibitory effect on SG formation in HPIV3-infected cells.

We previously showed that N and P were the critical components of IB formation of HPIV3 [7]. Because it is hard to reveal the formation of IBs during wild type HPIV3 infection due to lack of specific antibody against N or P, we used a recombinant virus HPIV3^HA-P^, (a HA tag fused to the N-terminal of P) to detect the expression of P and the formation of IBs in HPIV3^HA-P^-infected cells [45]. At 24–36 hpi, HA-P homogeneously distributed throughout the cytoplasm, and ~80% of cells formed SGs. (Fig 7G, panel 24 h and 36 h). As the time increased pi, HA-P gradually formed IBs, and staining with the antibody against HPIV3 also showed the IBs structures (Fig 7G, panel at 48 h and 60 h, cells marked with “+” represent the cells that form IBs). At 48 hpi, ~20% cells form IBs and SGs are always excluded in these IBs-containing cells; at 60 hpi, the number of IBs-containing cells increased up to ~40% and simultaneously, the number of SGs-containing cells decreased to ~20% (Fig 7G–7I). Similar results were also obtained when HEp-2 and MK2 cells were infected with HPIV3^HA-P^ (S6A and S6B Fig), suggesting that IBs have an inhibitory effect on SG formation. To confirm that HPIV3^HA-P^ has similar replication behavior, we also performed a complementary experiment in wild type HPIV3-infected cells and did find wild type HPIV3 induced IB formation in ~20% of cells at 48 hpi and ~40% of cells at 60 hpi (S6C Fig, cells marked with “+” represent the cells that form IBs, and S6D and S6E Fig). Similarly, SGs decreased along with the increase of IBs and all the cells containing IBs were deficient in SG formation. Taken together, these data show the inhibitory effect of IBs on SGs formation.

**Fig 8:**
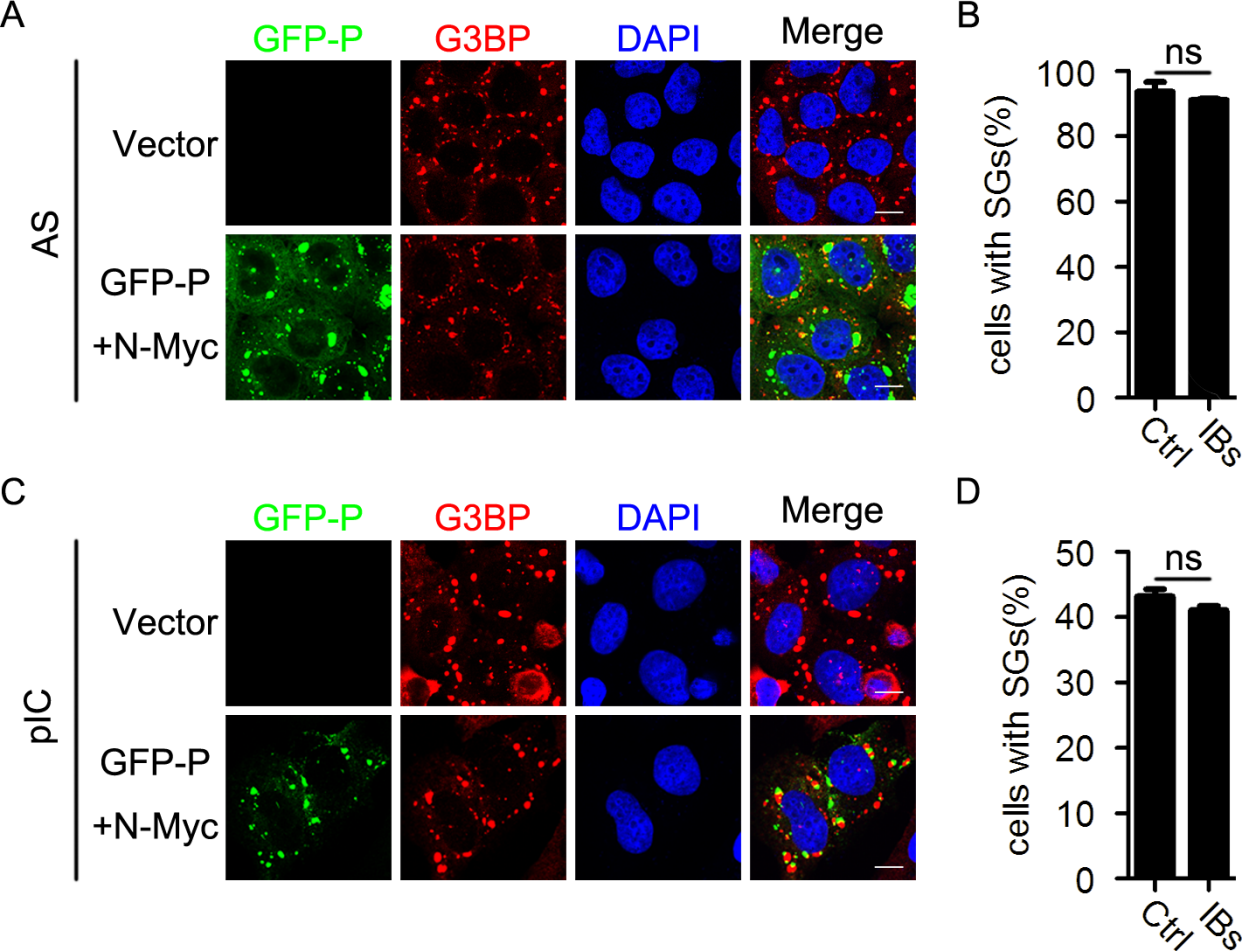
HPIV3 IBs do not inhibit SG formation induced by AS or pIC. (A-D) HeLa cells were transfected with an empty plasmid or co-transfected with plasmids encoding GFP-P and N-Myc for 24 h, then (A and B) treated with AS for another 1 h, or (C and D) transfected with pIC for another 12 h. (A and C) Cells were immunostained for G3BP (red). Nuclei were stained with DAPI (blue). The white scale bar corresponds to 10 μm. (B and D) The percentage of cells containing SGs was quantified in three independent experiments. Data are represented as means ±SD. Student’s t test: * P<0.05, ** P<0.01, *** P<0.001, ns = not significant.

Having established that IB formation can inhibit SG formation during HPIV3 infection, we next sought to determine whether the IBs formed by N and P of HPIV3 could inhibit SG formation induced by other stress stimuli. We over-expressed N and P of HPIV3 as well as AS or pIC stimuli and found that IB formation failed to inhibit AS- and pIC-induced SG formation (Fig 8A and 8C). The percentage of cells containing SGs was not affected in the presence of HPIV3 IBs (Fig 8B and 8D), suggesting that HPIV3 IBs do not disrupt typical stress response pathways of SG formation and inhibition of HPIV3-induced SG formation by HPIV3 IBs is specific.

### HPIV3 IBs inhibit SG formation by shielding newly synthesized viral RNAs

Our previous results have showed that IBs are the center of RNA synthesis [7]. Having found that mRNAs of HPIV3 induced the formation of SGs, which subsequently sequestered viral mRNAs to restrict viral replication, and that IBs inhibited SG formation in an HPIV3-infection specific manner, we sought to determine whether HPIV3 IBs could shield newly synthesized viral RNAs of HPIV3 to escape the inhibitory effect of SGs on viral replication.

**Fig 9:**
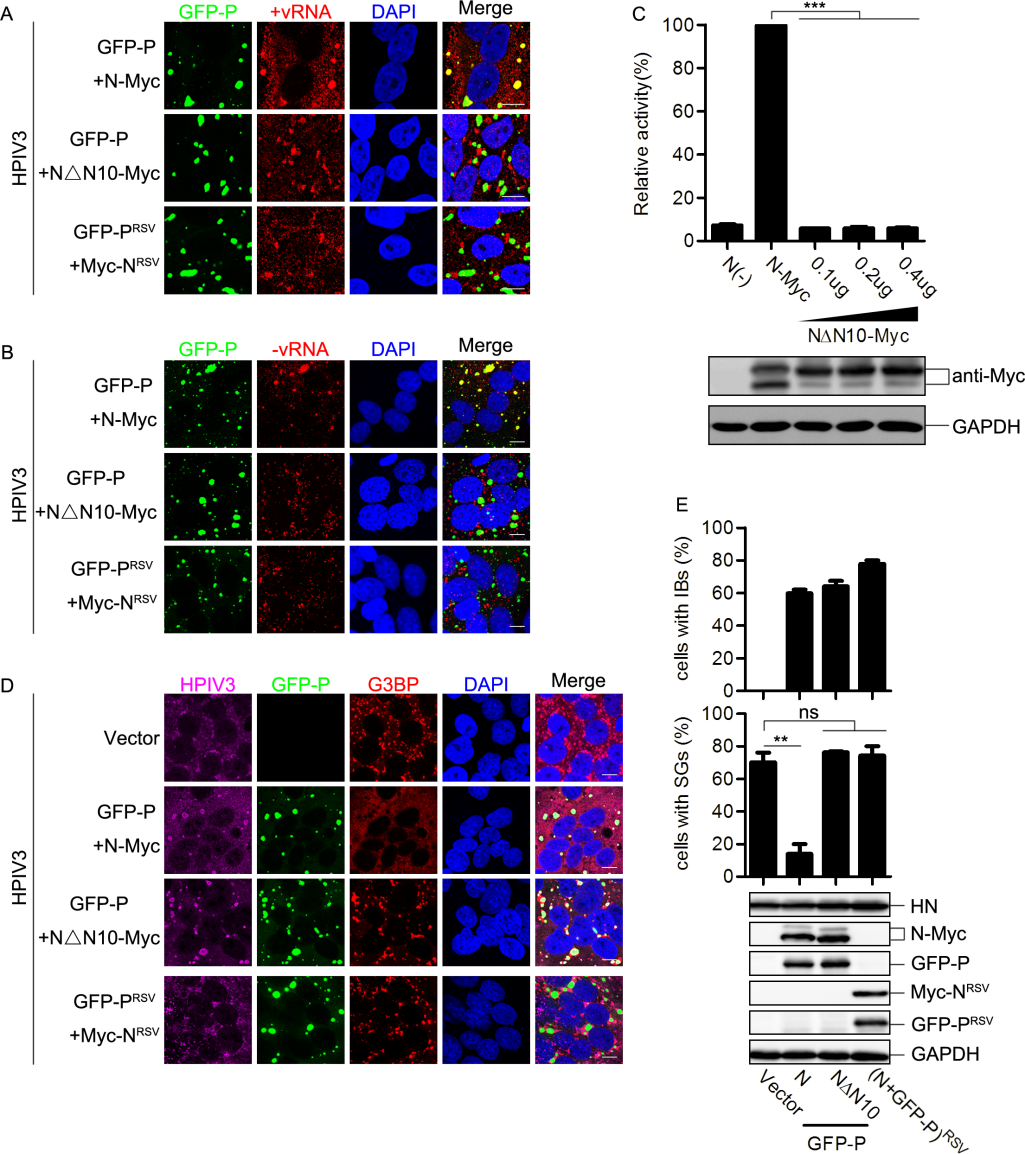
The inhibitory effect of HPIV3 IBs on SG formation is associated with the viral RNAs-holding capacity of the IBs. (A and B) HeLa cells were co-transfected with plasmids encoding GFP-P and N-Myc, GFP-P and NΔN10-Myc, or GFP-P^RSV^ and Myc-N^RSV^ for 24 h, then infected with HPIV3 for another 24 h. HPIV3 (A) +vRNAs (red) and (B)–vRNAs (red) were detected via RNA-FISH. The white scale bar corresponds to 10 μm. (C) HPIV3 mini-genome replicon system assay was performed and cell lysates were analyzed via western blot using anti-Myc and anti-GAPDH antibodies. (D and E) HeLa cells were transfected with an empty plasmid or co-transfected with plasmids encoding GFP-P and N-Myc, GFP-P and NΔN10-Myc, or GFP-P^RSV^ and Myc-N^RSV^ for 24 h, then infected with HPIV3 for another 24 h. (D) Cells were immunostained for HPIV3 (purple) and G3BP (red). Nuclei were stained with DAPI (blue). The white scale bar corresponds to 10 μm. (E) The percentage of cells containing IBs or SGs was quantified in three independent experiments. Cell lysates were analyzed via western blot using anti-HN, anti-Myc, anti-GFP and anti-GAPDH antibodies. Data are represented as means ±SD. Student’s t test: * P<0.05, ** P<0.01, *** P<0.001, ns = not significant.

We co-expressed GFP-P and N-Myc in HPIV3-infected cells and examined viral RNAs via RNA-FISH assay. The results revealed that both–vRNAs and +vRNAs obviously co-localized with IBs (Fig 9A and 9B, panel “GFP-P+ N-Myc”). To further demonstrate that the accumulation of viral RNAs in IBs is essential for the inhibition of SG formation, we identified a truncated mutant of N, NΔN10 (lacking N-terminal 10 amino acids of N), that could still form IBs when co-expressed with P, but was function-defective and failed to support viral RNA synthesis (Fig 9C). We found that neither -vRNAs nor +vRNAs co-localized with these function-defective IBs (Fig 9A and 9B, panel “GFP-P+ NΔN10-Myc”). Subsequently, IBs formed by GFP-P and NΔN10-Myc also failed to inhibit SG formation in HPIV3-infected cells (Fig 9D, panel “GFP-P+ NΔN10-Myc”), suggesting that viral RNAs which are synthesized and shielded in IBs are critical to avoid the induction of SG formation. Furthermore, previous research showed that co-expression of N and P of RSV can also form IBs [45]. Thus, we co-expressed GFP-P^RSV^ and Myc-N^RSV^ to form RSV IBs in HPIV3-infected cells and found that neither -vRNAs nor +vRNAs co-localized with RSV IBs (Fig 9A and 9B, panel “GFP-P^RSV^+ Myc-N^RSV^”). Again, RSV IBs were unable to inhibit HPIV3-induced SG formation (Fig 9D, panel “GFP-P^RSV^+ Myc-N^RSV^” and 9E). Taken together, these data show that HPIV3 IBs synthesized viral RNAs and simultaneously shielded viral RNAs to avoid the formation of SGs.

**Fig 10:**
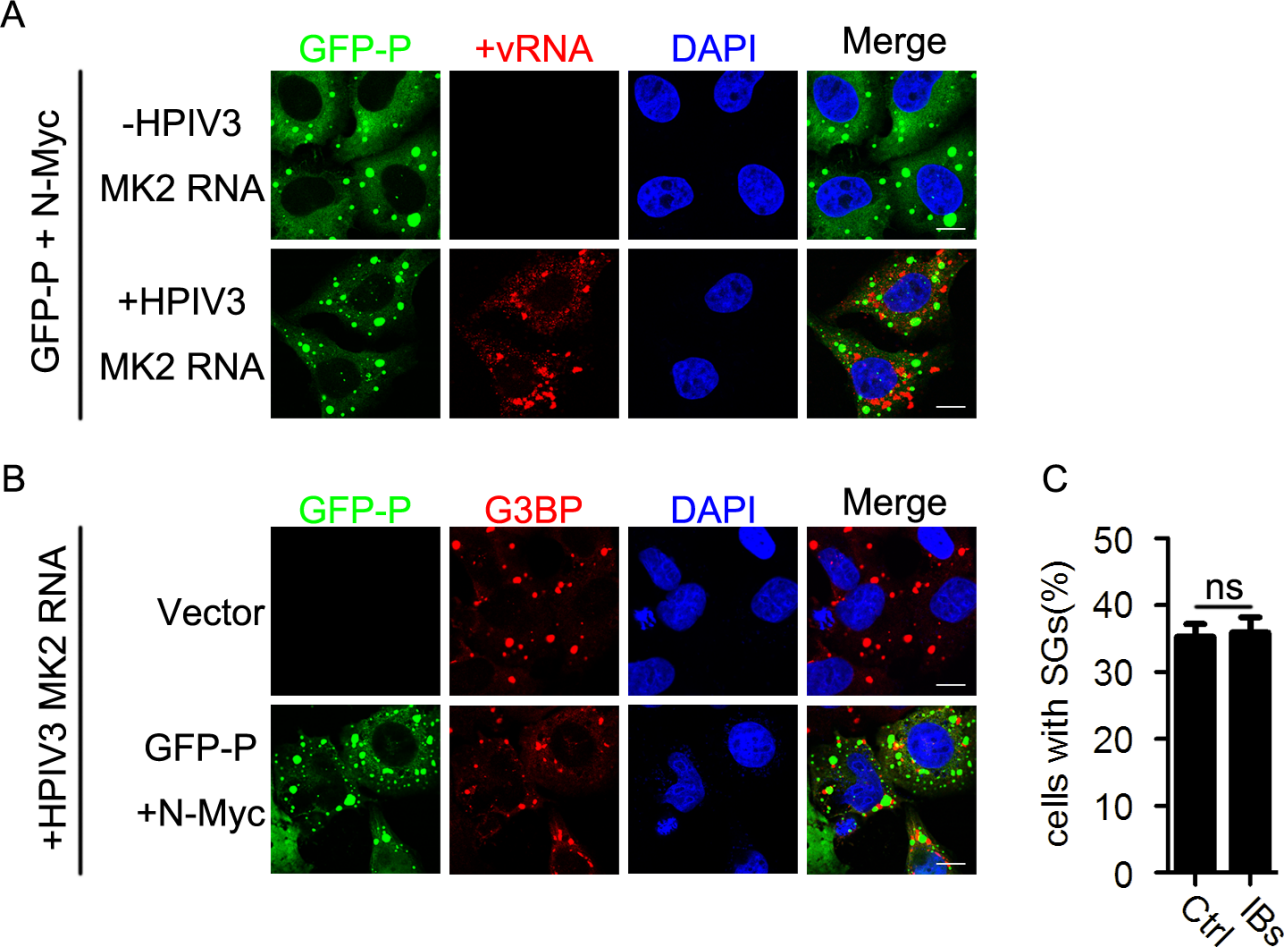
HPIV3 IBs do not inhibit the SG formation induced by transfection of HPIV3 viral RNAs. (A) HeLa cells were transfected with plasmids encoding GFP-P and N-Myc for 24 h, then transfected with RNA from HPIV3-infected MK2 cells for another 12 h. Cells were harvested, and HPIV3 +vRNA (red) was detected via RNA-FISH. The white scale bar corresponds to 10 μm. (B and C) HeLa cells were transfected with an empty plasmid or co-transfected with plasmids encoding GFP-P and N-Myc for 24 h, then transfected with RNA from HPIV3-infected MK2 cells for another 12 h. (B) Cells were immunostained for G3BP (red). Nuclei were stained with DAPI (blue). The white scale bar corresponds to 10 μm. (C) The percentage of cells containing SGs was quantified in three independent experiments. Data are represented as means ±SD. Student’s t test: * P<0.05, ** P<0.01, *** P<0.001, ns = not significant.

Since transfection of RNAs derived from HPIV3-infected cells was sufficient to induce SG formation, we next sought to determine whether HPIV3 IBs could inhibit the SG formation induced by the transfection of viral RNAs. We co-expressed N and P of HPIV3 as well as viral RNAs extracted from HPIV3-infected cells. As shown in the RNA-FISH assay, HPIV3 IBs neither captured +vRNAs of HPIV3 transfected into HeLa cells (Fig 10A, panel “+HPIV3 MK2 RNA”) nor suppressed SG formation induced by viral RNAs from HPIV3-infected cells (Fig 10B and 10C), suggesting that HPIV3 IBs specifically shield newly synthesized HPIV3 viral RNAs, thus resulting in the inhibition of HPIV3-induced SG formation.

## Discussion

In this study, we showed that mRNAs of HPIV3 induced SG formation in a PKR and eIF2α phosphorylation-dependent manner and found that HPIV3-induced SG formation is a general phenomenon in at least two cell types. Furthermore, we demonstrated that HPIV3 IBs efficiently suppressed the SG formation by shielding newly synthesized viral RNAs to escape recognition of PKR. Previous studies reported that some viruses-induced SGs were distinct from canonical SGs in both composition and function. Such as vesicular stomatitis virus (VSV) and rabies virus (RABV), two members of the *Rhabdoviridae* family, can trigger noncanonical SG formation in infected cells [36,37]. These SGs do not disassemble in the presence of CHX, a polysome-stabilizing drug that traps stalled mRNAs in polysomes, and VSV-induced pseudo-SGs lack several SG markers such as eIF3 and eIF4A. In our study, we observed that multiple canonical SG markers such as TIA-1, G3BP, eIF4A, eIF4E, and eIF4G, were all recruited into HPIV3-induced SGs (Fig 1 and S1 Fig). CHX treatment resulted in the disassembly of HPIV3- and AS-induced SGs (Fig 2), suggesting that HPIV3 infection induces canonical SG formation. But the disassembly of HPIV3-induced SGs in response to CHX is somewhat delayed compared to AS-induced SGs. Three possibilities may contribute to delayed disassembly of HPIV3-induced SGs. 1) HPIV3-induced SG formation is PKR dependent, but AS-induced SG formation is not (Fig 4A and 4B; S3C and S3D Fig); 2) HPIV3-induced SGs are rich of viral mRNAs, while AS-induced SGs are not (Fig 5A); 3) HPIV3-induced SGs go through a longer period of time during infection (24 h) than AS treatment (1.5 h) (Fig 2A–2D). Taken together, these differences may delay the disassembly of HPIV3-induced SGs in response to CHX.

Transfection of RNAs extracted from HPIV3-infected cells instead of the expression of each viral protein could induce PKR/eIF2α phosphorylation, thus resulting in SG formation in host cells (Fig 3A and 3B and S2A and S2B Fig), suggesting that vRNAs are the major factors triggering SG formation. Three major categories of vRNAs are generated during HPIV3 infection: viral genomic RNAs, anti-genomic RNAs and a series of PolyA^+^ RNAs. We isolated PolyA^+^ RNAs from the vRNAs and found that PolyA^+^ RNAs had a greater ability to induce SG formation than PolyA^-^ RNAs (Fig 3C and 3D). Since most PolyA^+^ RNAs within the vRNAs are viral mRNAs, we concluded that the mRNAs of HPIV3 are the major factors that induce SG formation during HPIV3 infection. But the exact mechanism how viral mRNAs activate phosphorylation of PKR and induce SG formation remains to be further explored.

Since SGs usually play inherent antiviral activity, what is the relationship between SGs and vRNAs during viral infection? In RABV- and Newcastle disease virus (NDV)-infected cells, vRNAs are synthesized in viral replication complexes (VRC), then +vRNAs, including viral mRNAs or/and anti-genomic RNAs, are selectively transported from VRC to SGs, whereas the viral genomic RNAs are excluded [37,43]; in IAV-infected cells, viral genomic RNAs localized within SGs [42], while in encephalomyocarditis virus (EMCV)-infected cells, viral dsRNAs are sequestered into SGs [41], suggesting that the vRNAs in SGs differ with viruses types. In this study, +vRNAs of HPIV3 were sequestered into HPIV3-induced SGs, while–vRNAs were enriched adjacent to and encircle the SGs (Fig 5A). Since SGs are considered storage sites of translational initiation trapped mRNAs, and viral mRNAs are the major component of +vRNAs, it is easy to understand that +vRNAs are selectively recruited into the core of HPIV3-induced SGs. Because in the process of HPIV3 replication, -vRNAs serve as the template for the synthesis of +vRNAs (include viral anti-genome RNA and mRNA),–vRNAs are closely coupling with +vRNAs and encircle SGs, suggesting that HPIV3-induced SGs can capture viral mRNAs and may restrict viral replication through inhibition of viral translation.

HPIV3 infection induces SG formation in a PKR-dependent manner. Knockdown of PKR expression significantly inhibits eIF2α phosphorylation, thus blocking SG formation (Fig 4), which is in accordance with the critical role of PKR in SG formation triggered by many viruses [20,28,42,46]. Furthermore, knockdown of PKR expression increases HPIV3 protein expression and viral production (Fig 4E and 4F), implying that HPIV3-induced SGs have an antiviral role. We further disrupted SG formation via knockdown of G3BP expression and over-

Inhibition of SG formation via expression of an eIF2α non-phosphorylatable mutant, eIF2α-S51A, dramatically increased viral protein expression and viral production of HPIV3 (Fig 5), but had no effect on the production of IFN (Figs 5 and 6), suggesting that HPIV3-induced SGs indeed play an antiviral role in spite of the IFN induction. These results are consistent with previous findings that SGs are dispensable for induction of innate immune response [38,44].

How does HPIV3 escape from antiviral activity of SGs? Some viruses appear to inhibit SG formation during infection [31,34,47–56], and some viruses may tolerate or exploit SGs to facilitate their own replication [37,57,58]. In our study, we showed that N or P expressed individually could form viral IBs in some HPIV3-infected cells, and all these IB-containing cells were devoid of SG formation (Fig 7A–7C), suggesting that HPIV3 IBs have a novel inhibitory effect on SG formation. Our previous work showed that over-expression of HPIV3 N and P proteins can form IBs, while a point mutant of N, N_L478A_, failed to interact with P, thus, resulting in failure to form IBs when co-expressed with P [7]. Therefore, we co-expressed P with N or N_L478A_ to detect their inhibitory effect on SG formation. Our results showed that N and P formed large numbers of IBs which efficiently inhibited SG formation, while N_L478A_ failed to form IBs with P and did not inhibit SG formation (Fig 7D–7F). We confirmed this result by infecting cells with HPIV3^HA-P^ and found that all the HPIV3^HA-P^-infected cells containing IBs were deficient in SG formation (Fig 7G–7I and S6A–S6B Fig), suggesting that HPIV3 IBs inhibit SG formation during HPIV3 infection. It should be noted that IB formation was not observed prior to approximately 48 hpi in HPIV3^HA-P^-infected cells, which is later than IB formation prior to 24 hpi as described in our previous studies [45]. The possible reason for the difference is that a new batch of cell lines were used in this assay.

Previous studies showed that interplay between viral IBs and SGs is complicated, and there is no common conclusion of how viral IBs influence SG formation. RABV-induced SGs located closely to viral factories which are IB-like structures, termed as Negri bodies (NBs), and viral RNAs are synthesized in NBs and viral mRNAs are specially transported from NBs to SGs [37]; VSV-induced SG-like structures co-localize or even share the same structure with viral cytoplasmic IBs [36]; IBs of RSV sequester p38 and OGT into viral IBs to suppress SG assembly [32,57]; EBOV sequesters many SG maker proteins to form SG-like structures inside viral IBs, probably resulting in the inhibition of antiviral role of SGs [34].

How do HPIV3 IBs inhibit SG formation during HPIV3 infection? We found that SGs still robustly formed in IB-containing cells once the cells were treated with AS or pIC (Fig 8), suggesting that HPIV3 IBs do not inhibit AS- or pIC-induced SG formation and the inhibition of HPIV3-induced SG formation by IBs is virus-specific. Since IBs are the sites for RNA synthesis, we then sought to determine whether HPIV3 IBs could shield viral RNAs of HPIV3 to inhibit SG formation. Heinrich BS, et al. showed that VSV synthesizes its primary viral RNA throughout the cell cytoplasm by input RNP in the absence of viral IBs. Accumulation of viral proteins results in the formation of viral IBs that contain the RNA synthesis machine and become the predominant sites of viral RNA synthesis[59]. It is likely that this model also applies to HPIV3 infection in which the synthesis of viral RNA is prior to the formation of IBs and is initially unrestricted, but subsequently is shielded to avoid the SG formation. We then evaluated the vRNA-holding capacity of HPIV3 IBs and found that IBs formed by HPIV3 N and P redirect viral RNA synthesis from cytoplasm into IBs which shield viral RNAs and avoid the formation of SGs (Fig 9). However, IBs formed by RSV N and P failed to shield viral RNAs of HPIV3 and were therefore unable to inhibit HPIV3-induced SG formation (Fig 9). Similarly, IBs formed by NΔN10 and P of HPIV3 were replication-deficient and unable to shield viral RNAs or inhibit HPIV3-induced SG formation (Fig 9), suggesting that the vRNAs-holding capacity is rather critical for HPIV3 IBs to inhibit SG formation. Furthermore, to our surprise, we found that HPIV3 IBs also failed to capture the transfected vRNAs extracted from HPIV3-infected cells, suggesting that IBs of HPIV3 only specifically shield newly synthesized viral RNAs to inhibit SG formation (Fig 10). It is conceivable that HPIV3 IBs synthesize and hold viral mRNAs temporarily, then release viral mRNAs slowly or in an unknown way, which avoid accumulation of mRNAs instantly and SG formation. However, the exact mechanism how IBs shield newly synthesized viral RNAs and escape from recognition of PKR need to be further explored.

To our knowledge, this is first report to describe a novel inhibitory effect of IBs on virus-induced SG formation by specifically capturing its own newly synthesized viral RNAs, thus helping the virus to escape antiviral SG formation and facilitate its own replication.

## Materials and methods

### Cells, viruses, and reagents

HeLa (Human cervical cancer epithelial cells and were obtained from China Center for Type Culture Collection), HeLa-GFP-G3BP (stable GFP-G3BP expression, derived from HeLa), LLC-MK2 (monkey kidney cell line and were obtained from China Center for Type Culture Collection), HEp-2 (Human laryngeal carcinoma epithelial cells and were originally obtained from American Type Culture Collection), HEK293T (Human embryonic kidney 293 cells and were obtained from China Center for Type Culture Collection), RIG-I^-/-^ and VISA^-/-^ MEF (mouse embryonic fibroblast cells and obtained from Shu HB lab, Wuhan university, China) cells were cultured in Dulbecco’s modified Eagle’s medium (DMEM, Gibco) supplemented with 10% fetal bovine serum (FBS, Gibco) and 1% penicillin-streptomycin (Gibco). PKR or G3BP stable knockdown cells were made via transduction with lentiviral shRNA transduction particles. Knockdown cells were cultured in DMEM maintenance media with puromycin (1 ug/ml, Sigma). Recombinant HPIV3 carrying an HA tag fused with the N-terminal of viral P (HPIV3^HA-P^) was constructed by our laboratory as described previously [45]. Both HPIV3 and recombinant HPIV3^HA-P^ were propagated in LLC-MK2 cells by inoculation at a multiplicity of infection (MOI) of 0.1. AS (Sigma) and CHX (MCE) were used at concentrations of 0.5 mM and 100ug/ml, respectively, for the indicated times. pIC was purchased from InvivoGen.

### Virus infection and plaque assay

Cells were cultured in 6-well or 24-well plates at a density of 70%-80% at 37°C overnight and incubated with HPIV3 at an MOI of 1 plaque-forming unit/cell for 2h at 37°C and 5% CO_2_. Then the medium was replaced with fresh medium with 10% FBS. For the plaque assay, virus stock was serially diluted 10-fold up to 10^5^, and MK2 cells grown in 24-well plates were infected with 400 ul of the dilutions for 2h at 37°C and 5% CO_2_. Then, the medium was replaced with methylcellulose, and the cell plates were incubated at 37°C and 5% CO_2_ for an additional 3–4 days until visible viral plaque was detected. After being stained with crystal violet, the plaques were counted to calculate the viral titers.

### Western blot analysis

Cells were harvested and lysed with lysis buffer (150 mM NaCl, 50 mM Tris-HCl [pH 7.4], 1% Triton X-100, 1 mM EDTA [pH 8.0] and 0.1% sodium dodecyl sulfate [SDS]) for 30 min on ice. The supernatants were collected via centrifugation at 12000 g at 4°C for 30 min. The protein concentration was determined using the Bradford assay method (Bio-Rad). Samples were boiled with SDS-PAGE loading buffer at 100°C for 10min and resolved via 10% SDS polyacrylamide gel electrophoresis (SDS-PAGE). Proteins were then transferred to nitrocellulose membranes. The membrane was blocked with 5% milk in phosphate buffered saline (PBS) with 0.1% Tween 20 (PBST) for 1h before being incubated with primary antibodies overnight and then incubated with secondary antibodies for another 1h. The primary antibodies used were as follows: mouse anti-HN (1:1000, Abcam), mouse anti-GAPDH (1:2500, Santa Cruz), rabbit anti-eIF2α (1:1000, CST), rabbit anti-phosphorylated eIF2α (1:1000, CST), rabbit anti-PKR (1:1000, Abcam), rabbit anti-phosphorylated PKR (1:1000, Abcam), mouse anti-G3BP (1:1000, BD Biosciences), mouse anti-HA tag (1:10000, Sigma), rabbit anti-IRF3 (1:1000, Abcam), and mouse anti-LMNB1 (1:1000, Applygen). HRP-conjugated goat anti-mouse immunoglobulin (IgG) (1:5000) and goat anti-rabbit IgG (1:5000) were used as secondary antibodies.

### Immunofluorescence analysis

HeLa cells were cultured on coverslips in 24-well plates overnight. After transfection or/and infection, cells were harvested at the indicated times. Cells were fixed with 4% paraformaldehyde and permeabilized with 0.2% Triton X-100 for 20min at room temperature. After being blocked with 3% bovine serum albumin (BSA) for 30min, cells were incubated with primary antibodies diluted in 1% BSA at 4°C overnight and secondary antibodies diluted in 1% BSA at room temperature for another 1h. Cells were mounted with Fluoroshield (Sigma) and examined by using a Leica confocal microscope after staining with 1 mg/ml 4’,6-diamidino-2-phenylindole (DAPI) in PBS. The primary antibodies used were as follows: goat anti-TIA-1 (1:200, Santa Cruz), goat anti-HPIV3 (1:1000, Abcam), rabbit anti-TIA-1 (1:500, ABclonal), rabbit anti-G3BP (1:500, ABclonal), rabbit anti-eIF4A (1:500, ABclonal), rabbit anti-eIF4E (1:500, ABclonal), rabbit anti-eIF4G (1:200, CST), rabbit anti-phosphorylated eIF2α (1:200, CST), mouse anti-G3BP (1:500, BD Bioscience), mouse anti-HA tag (1:2000, Sigma), mouse anti-Flag tag (1:1000, Sigma), and mouse anti-Myc tag (1:200, Santa Cruz). The secondary antibodies used were as follows: Alexa Fluor 647 donkey anti-goat IgG (1:1000, Invitrogen), Alexa Fluor 488 donkey anti-rabbit IgG (1:1000, Invitrogen), and Alexa Fluor 594 donkey anti-mouse IgG (1:1000, Invitrogen).

### RNA-FISH

The RNA-FISH assay was performed according to the manufacturer’s instructions for the QuantiGene ViewRNA ISH Cell Assay kit (Affymetrix). Cells were fixed in 4% paraformaldehyde solution at room temperature for 30 min and permeabilized with detergent solution at room temperature for 5 min. Protease solution was added to the cells at a suitable dilution (1:1000–1:4000) in PBS, and the cells were incubated at room temperature for 10 min. After being washed with PBS, the cells were incubated with a special probe set at a 1:100 dilutions at 40°C for 3 h. Then a pre-amplifier, amplifier, and label probe were sequentially added to the cells all at a 1:25 dilution, and the cells were incubated at 40°C for 30 min. Cells were mounted with Fluoroshield (Sigma) and examined by using a Leica confocal microscope after being stained with 1 mg/ml DAPI in PBS. The probe sets targeted to HPIV3 +vRNA and HPIV3 –vRNA were purchased from Affymetrix.

### RNA preparation and transfection

Total RNA from mock- or HPIV3-infected cells was solated using TRIzol reagent and precipitated by isopropanol. PolyA^+^ RNA was subsequently isolated from the total RNA according to the manufacturer’s instructions for the GenElute mRNA Miniprep Kit (Sigma). We added 15 ul oligo (dT) polystyrene beads to 500 ug total RNA samples. The mixture was incubated at 70°C for 10 min and at room temperature for another 10 min. After centrifugation, supernatants were subjected to ethanol precipitation to obtain a concentrated PolyA^-^ RNA fraction. The beads were washed twice with 500 ul wash solution through a GenElute spin filter. The PolyA^+^ RNA fraction was subsequently eluted and collected. Purification was repeated twice to yield a pure PolyA^+^ RNA fraction. cDNA of HPIV3 N was constructed into PBS vector downstream of T7 promoter and template was linearized and purified by phenol: chloroform and ethanol precipitation. mRNA was transcribed *in vitro* according to the manufacturer’s instructions for the Transcript Aid T7 High Yield Transcription Kit (Thermo Scientific) and purified using the RNeasy Mini Kit (QIGEN). We transfected 0.5 ug of the RNA samples into HeLa cells in 24 wells using Lipofectamine 2000 reagent (invitrogen). At 12 h post-transfection, the cells were harvested and subjected to immunofluorescence analysis.

### Lentiviral transduction of exogenous gene

HEK293T cells were transfected with pWPI-GFP-G3BP and the package plasmids pCMV-VSV-G, pCMV-Tat and pCMV-R8.91. At 48 h post-transfection, supernatants containing lentiviruses were collected to infect HeLa cells. Then the HeLa cells stably expressing GFP-G3BP were passaged. The shRNA lentiviruses were packaged via transfection of PKR shRNA or G3BP shRNA with the package plasmids psPAX2 and pMD2.G into HEK293T cells. At 48 h post-transfection, lentiviruses were collected to infect HeLa cells to induce stable knockdown of PKR or G3BP expression.

### Nuclear-cytosol extraction

Cells were harvested, and then the nuclei fraction and the cytosol fraction were separated and extracted according to the manufacturer’s instructions for the Nuclear-Cytosol Extraction kit (Applygen). Samples were boiled with SDS-PAGE loading buffer for western blot analysis.

### Luciferase assay

HEK 293T cells were transfected with 50 ng IFNβ-Luc reporter and 20 ng TK-Luc reporter for 12 h and then infected with HPIV3 for another 24 h. The firefly luciferase activity was assayed and normalized by that of Renilla luciferase. Experiments were performed in triplicate.

### qPCR assay

Total RNA were isolated for qPCR analysis to measure the indicated RNA abundance. Data shown are the relative abundance of the indicated RNA normalized to that of GAPDH. The following Primers were used:

HPIV3 M protein forward: 5’-AGAAGAACAGTCAAAGCGAAAG-3’;

HPIV3 M protein reverse: 5’-CTCCAACTAATCCCAAAG-3’;

Human GAPDH forward: 5’-GAGTCAACGGATTTGGTCGT-3’;

Human GAPDH reverse: 5’-GACAAGCTTCCCGTTCTCAG-3’;

Mouse IFNB1 forward: 5’-AGTTACACTGCCTTTGCC-3’;

Mouse IFNB1 reverse: 5’-TGAGGACATCTCCCACGT-3’;

Mouse GAPDH forward: 5’-GCATTGTGGAAGGGCTCA-3’;

Mouse GAPDH reverse: 5’-AGGCGGCACGTCAGATC-3’.

### HPIV3 mini-genome replicon system assay

HeLa cells were infected with vTF7-3 for 1h, then transfected with pGADT7-P (125ng), pGEM4-L (100ng), HPIV3 minigenome plasmid carrying the luciferase reporter gene (50ng) together with pCDNA3.0-N-Myc (100ng) or pCDNA3.0-NΔN10-Myc (100ng, 200ng or 400ng) for 24h. Cell lysates were collected to determine the luciferase activity or analyzed via western blot.

## Supporting information

S1 Fig HPIV3-triggered SG formation is a general process. (A) HeLa cells were infected with HPIV3 (MOI = 1) for 24 h. Cells were immunostained for HPIV3 (purple), eIF4A/4E/4G (green), and G3BP (red). Nuclei were stained with DAPI (blue). The white scale bar corresponds to 10μm. (B) HEp-2 cells were infected with HPIV3 (MOI = 1), and MK2 cells were infected with HPIV3 (MOI = 0.1) for 24h. Cells were immunostained for HPIV3 (purple), TIA-1 (green), and G3BP (red). Nuclei were stained with DAPI (blue). The white scale bar corresponds to 10μm. (C) HeLa cells were mock-treated, infected with HPIV3 (MOI = 1) for 24h, or treated with AS (0.5 mM) for 1h. Cells were immunostained for HPIV3 (purple), phosphorylated eIF2α (green), and G3BP (red). Nuclei were stained with DAPI (blue). The white scale bar corresponds to 10μm.
(TIF)

S2 Fig Over-expression of HPIV3 viral proteins fails to induce SG formation and the time course of SG formation induced by RNA transfection. (A and B) HeLa cells were transfected with an empty plasmid or plasmids encoding N, P, M, F, or HN for 24 h or treated with AS for 1 h. (A) Cells were immunostained for G3BP (green) and Myc/HA/Flag tag (viral protein, red). Nuclei were stained with DAPI (blue). The white scale bar corresponds to 10μm. (B) Cell lysates were analyzed via western bot using anti-Myc, anti-HA, anti-Flag, anti-phosphorylated eIF2α, anti-eIF2α, and anti-GAPDH antibodies. (C and D) HeLa cells were transfected with the indicated RNA samples from HPIV3 infected MK2 cells. (C) Cells were immunostained for TIA-1 (green) and G3BP (red). Nuclei were stained with DAPI (blue). The white scale bar corresponds to 10μm. (D) The percentage of cells containing SGs was quantified in three independent experiments. (E) *In vitro* transcribed HPIV3 N mRNA was transfected into HeLa cells. Cells were immunostained for TIA-1 (green) and G3BP (red). Nuclei were stained with DAPI (blue).
Data are represented as means ±SD. Student’s t test: * P<0.05, ** P<0.01, *** P<0.001, ns = not significant.
(TIF)

S3 Fig Inhibition of SG formation induced by pIC or AS. (A-D) HeLa cells with or without PKR knockdown were transfected with pIC for 12 h or treated with AS (0.5 mM) for 1 h. (A and C) Cells were immunostained for TIA-1 (green) and G3BP (red). Nuclei were stained with DAPI (blue). The white scale bar corresponds to 10μm. (B and D) The percentage of cells containing SGs was quantified in three independent experiments. (E and F) HeLa cells with or without G3BP knockdown were treated with AS (0.5 mM) for 1 h. (E) Cells were immunostained for TIA-1 (green) and G3BP (red). Nuclei were stained with DAPI (blue). The white scale bar corresponds to 10μm. (F) The percentage of cells containing SGs was quantified in three independent experiments. (G and H) HeLa cells were transfected with an empty plasmid or plasmids encoding eIF2α or the nonophosphorylatable mutant eIF2α-S51A for 24 h, then treated with AS (0.5 mM) for another 1 h. (G) Cells were immunostained for G3BP (green) and HA (red). Nuclei were stained with DAPI (blue). The white scale bar corresponds to 10μm. (H) The percentage of cells containing SGs was quantified in three independent experiments. Data are represented as means ±SD. Student’s t test: * P<0.05, ** P<0.01, *** P<0.001, ns = not significant.
(TIF)

S4 Fig IFN induction is not required for SG formation. (A) HeLa cells were transfected with an empty plasmid or plasmids encoding RIG-I-N or VISA for 24 h or pIC for 12 h. Cells were immunostained for TIA-1 (purple), G3BP (green) and Flag (red). Nuclei were stained with DAPI (blue). The white scale bar corresponds to 10 μm. (B) HEK293T cells were transfected with 50 ng IFNβ-Luc reporter and 20 ng TK-Luc reporter together with the indicated plasmid encoding Flag-RIG-I-N or Flag-VISA or pIC for 24 h. Cells were harvested for a luciferase assay. Cell lysates were analyzed via western blot using anti-Flag and anti-GAPDH antibodies. (C-E) Wide type, RIG-I^-/-^ or VISA^-/-^ MEF cells were infected with HPIV3 (MOI = 1) for 24 h. (C) Cells were immunostained for HPIV3 (purple), TIA-1 (green) and G3BP (red). Nuclei were stained with DAPI (blue). The white scale bar corresponds to 10 μm. (D) The percentage of cells containing SGs was quantified in three independent experiments. (E) Total RNA were isolated for qPCR to determine the IFNβ mRNA abundance and normalized to that of GAPDH. Data are represented as means ±SD. Student’s t test: * P<0.05, ** P<0.01, *** P<0.001, ns = not significant.
(TIF)

S5 Fig Over-expression of viral proteins fails to inhibit HPIV3-triggered SG formation. (A and B) HeLa cells were transfected with an empty plasmid or plasmids encoding M, F, or HN for 24 h, then infected with HPIV3 (MOI = 1) for another 24h. (A) Cells were immunostained for HPIV3 (purple), G3BP (green), and Myc/HA/Flag tag (viral protein, red). Nuclei were stained with DAPI (blue). The white scale bar corresponds to 10 μm. (B) The percentage of cells containing SGs was quantified in three independent experiments. Cell lysates were analyzed via western blot using anti-Myc, anti-Flag, anti-HA and anti-GAPDH antibodies. (C and D) HeLa cells were transfected with an empty plasmid or plasmids encoding N-Myc or HA-P or co-transfected with plasmids encoding GFP-P together with N-Myc or N_L478A_-Myc for 24 h, then mock infected or infected with HPIV3 (MOI = 1) for another 24 h. Cells lysates were analyzed using anti-HN, anti-Myc, anti-HA, anti-GFP and anti-GAPDH antibodies. Data are represented as means ±SD. Student’s t test: * P<0.05, ** P<0.01, *** P<0.001, ns = not significant.
(TIF)

S6 Fig HPIV3 IBs inhibit SG formation. (A) HEp-2 cells were infected with HPIV3^HA-P^ (MOI = 10), and (B) MK2 cells were infected with HPIV3^HA-P^ (MOI = 1). At the indicated time points pi, cells were immunostained for HPIV3 (purple), HA (green), and G3BP (red). Nuclei were stained with DAPI (blue). The white scale bar corresponds to 10 μm. (C-E) HeLa cells were infected with HPIV3 (MOI = 1). (C) At the indicated time points pi, cells were immunostained for HPIV3 (purple), TIA-1 (green), and G3BP (red). Nuclei were stained with DAPI (blue). The white scale bar corresponds to 10 μm. (D and E) The percentage of cells containing IBs or SGs was quantified in three independent experiments. Data are represented as means ±SD. Student’s t test: * P<0.05, ** P<0.01, *** P<0.001, ns = not significant.
(TIF)

## References

[1] Glezen WP, Frank AL, Taber LH, Kasel JA (1984) Parainfluenza virus type 3: seasonality and risk of infection and reinfection in young children. J Infect Dis 150: 851–857. 6094674 10.1093/infdis/150.6.851

[2] Banerjee AK, Barik S, De BP (1991) Gene expression of nonsegmented negative strand RNA viruses. Pharmacol Ther 51: 47–70. 1771177 10.1016/0163-7258(91)90041-j

[3] Ebata SN, Cote MJ, Kang CY, Dimock K (1991) The fusion and hemagglutinin-neuraminidase glycoproteins of human parainfluenza virus 3 are both required for fusion. Virology 183: 437–441. 1647076 10.1016/0042-6822(91)90162-5

[4] Galinski MS (1991) Paramyxoviridae: transcription and replication. Adv Virus Res 39: 129–162. 2038953 10.1016/s0065-3527(08)60794-0

[5] Hoffman MA, Banerjee AK (2000) Precise mapping of the replication and transcription promoters of human parainfluenza virus type 3. Virology 269: 201–211. doi: 10.1006/viro.2000.0223 10725212 10.1006/viro.2000.0223

[6] Durbin AP, Siew JW, Murphy BR, Collins PL (1997) Minimum protein requirements for transcription and RNA replication of a minigenome of human parainfluenza virus type 3 and evaluation of the rule of six. Virology 234: 74–83. doi: 10.1006/viro.1997.8633 9234948 10.1006/viro.1997.8633

[7] Zhang S, Chen L, Zhang G, Yan Q, Yang X, et al (2013) An amino acid of human parainfluenza virus type 3 nucleoprotein is critical for template function and cytoplasmic inclusion body formation. J Virol 87: 12457–12470. doi: 10.1128/JVI.01565-13 24027324 10.1128/JVI.01565-13 PMC3807885

[8] Zhang G, Zhong Y, Qin Y, Chen M (2015) Interaction of Human Parainfluenza Virus Type 3 Nucleoprotein with Matrix Protein Mediates Internal Viral Protein Assembly. J Virol 90: 2306–2315. doi: 10.1128/JVI.02324-15 26656716 10.1128/JVI.02324-15 PMC4810691

[9] Zhang G, Zhang S, Ding B, Yang X, Chen L, et al (2014) A leucine residue in the C terminus of human parainfluenza virus type 3 matrix protein is essential for efficient virus-like particle and virion release. J Virol 88: 13173–13188. doi: 10.1128/JVI.01485-14 25187547 10.1128/JVI.01485-14 PMC4249104

[10] Akira S, Uematsu S, Takeuchi O (2006) Pathogen recognition and innate immunity. Cell 124: 783–801. doi: 10.1016/j.cell.2006.02.015 16497588 10.1016/j.cell.2006.02.015

[11] White JP, Lloyd RE (2012) Regulation of stress granules in virus systems. Trends Microbiol 20: 175–183. doi: 10.1016/j.tim.2012.02.001 22405519 10.1016/j.tim.2012.02.001 PMC3322245

[12] Hornung V, Ellegast J, Kim S, Brzozka K, Jung A, et al (2006) 5'-Triphosphate RNA is the ligand for RIG-I. Science 314: 994–997. doi: 10.1126/science.1132505 17038590 10.1126/science.1132505

[13] Pichlmair A, Schulz O, Tan CP, Naslund TI, Liljestrom P, et al (2006) RIG-I-mediated antiviral responses to single-stranded RNA bearing 5'-phosphates. Science 314: 997–1001. doi: 10.1126/science.1132998 17038589 10.1126/science.1132998

[14] Kato H, Takeuchi O, Mikamo-Satoh E, Hirai R, Kawai T, et al (2008) Length-dependent recognition of double-stranded ribonucleic acids by retinoic acid-inducible gene-I and melanoma differentiation-associated gene 5. J Exp Med 205: 1601–1610. doi: 10.1084/jem.20080091 18591409 10.1084/jem.20080091 PMC2442638

[15] Schlee M, Roth A, Hornung V, Hagmann CA, Wimmenauer V, et al (2009) Recognition of 5' triphosphate by RIG-I helicase requires short blunt double-stranded RNA as contained in panhandle of negative-strand virus. Immunity 31: 25–34. doi: 10.1016/j.immuni.2009.05.008 19576794 10.1016/j.immuni.2009.05.008 PMC2824854

[16] Kato H, Takeuchi O, Sato S, Yoneyama M, Yamamoto M, et al (2006) Differential roles of MDA5 and RIG-I helicases in the recognition of RNA viruses. Nature 441: 101–105. doi: 10.1038/nature04734 16625202 10.1038/nature04734

[17] Xu LG, Wang YY, Han KJ, Li LY, Zhai Z, et al (2005) VISA is an adapter protein required for virus-triggered IFN-beta signaling. Mol Cell 19: 727–740. doi: 10.1016/j.molcel.2005.08.014 16153868 10.1016/j.molcel.2005.08.014

[18] Seth RB, Sun L, Ea CK, Chen ZJ (2005) Identification and characterization of MAVS, a mitochondrial antiviral signaling protein that activates NF-kappaB and IRF 3. Cell 122: 669–682. doi: 10.1016/j.cell.2005.08.012 16125763 10.1016/j.cell.2005.08.012

[19] Kawai T, Takahashi K, Sato S, Coban C, Kumar H, et al (2005) IPS-1, an adaptor triggering RIG-I- and Mda5-mediated type I interferon induction. Nat Immunol 6: 981–988. doi: 10.1038/ni1243 16127453 10.1038/ni1243

[20] Garcia MA, Meurs EF, Esteban M (2007) The dsRNA protein kinase PKR: virus and cell control. Biochimie 89: 799–811. doi: 10.1016/j.biochi.2007.03.001 17451862 10.1016/j.biochi.2007.03.001

[21] Nallagatla SR, Hwang J, Toroney R, Zheng X, Cameron CE, et al (2007) 5'-triphosphate-dependent activation of PKR by RNAs with short stem-loops. Science 318: 1455–1458. doi: 10.1126/science.1147347 18048689 10.1126/science.1147347

[22] Kedersha N, Chen S, Gilks N, Li W, Miller IJ, et al (2002) Evidence that ternary complex (eIF2-GTP-tRNA(i)(Met))-deficient preinitiation complexes are core constituents of mammalian stress granules. Mol Biol Cell 13: 195–210. doi: 10.1091/mbc.01-05-0221 11809833 10.1091/mbc.01-05-0221 PMC65082

[23] Kimball SR, Horetsky RL, Ron D, Jefferson LS, Harding HP (2003) Mammalian stress granules represent sites of accumulation of stalled translation initiation complexes. Am J Physiol Cell Physiol 284: C273–284. doi: 10.1152/ajpcell.00314.2002 12388085 10.1152/ajpcell.00314.2002

[24] Kedersha NL, Gupta M, Li W, Miller I, Anderson P (1999) RNA-binding proteins TIA-1 and TIAR link the phosphorylation of eIF-2 alpha to the assembly of mammalian stress granules. J Cell Biol 147: 1431–1442. 10613902 10.1083/jcb.147.7.1431 PMC2174242

[25] Gallouzi IE, Brennan CM, Stenberg MG, Swanson MS, Eversole A, et al (2000) HuR binding to cytoplasmic mRNA is perturbed by heat shock. Proc Natl Acad Sci U S A 97: 3073–3078. 10737787 10.1073/pnas.97.7.3073 PMC16194

[26] McCormick C, Khaperskyy DA (2017) Translation inhibition and stress granules in the antiviral immune response. Nat Rev Immunol. 10.1038/nri.2017.63 28669985

[27] Iseni F, Garcin D, Nishio M, Kedersha N, Anderson P, et al (2002) Sendai virus trailer RNA binds TIAR, a cellular protein involved in virus-induced apoptosis. Embo j 21: 5141–5150. doi: 10.1093/emboj/cdf513 12356730 10.1093/emboj/cdf513 PMC129035

[28] Okonski KM, Samuel CE (2013) Stress granule formation induced by measles virus is protein kinase PKR dependent and impaired by RNA adenosine deaminase ADAR1. J Virol 87: 756–766. doi: 10.1128/JVI.02270-12 23115276 10.1128/JVI.02270-12 PMC3554044

[29] Lu Y, Wambach M, Katze MG, Krug RM (1995) Binding of the influenza virus NS1 protein to double-stranded RNA inhibits the activation of the protein kinase that phosphorylates the elF-2 translation initiation factor. Virology 214: 222–228. 8525619 10.1006/viro.1995.9937

[30] Talon J, Horvath CM, Polley R, Basler CF, Muster T, et al (2000) Activation of interferon regulatory factor 3 is inhibited by the influenza A virus NS1 protein. J Virol 74: 7989–7996. 10933707 10.1128/jvi.74.17.7989-7996.2000 PMC112330

[31] White JP, Cardenas AM, Marissen WE, Lloyd RE (2007) Inhibition of cytoplasmic mRNA stress granule formation by a viral proteinase. Cell Host Microbe 2: 295–305. doi: 10.1016/j.chom.2007.08.006 18005751 10.1016/j.chom.2007.08.006

[32] Fricke J, Koo LY, Brown CR, Collins PL (2013) p38 and OGT sequestration into viral inclusion bodies in cells infected with human respiratory syncytial virus suppresses MK2 activities and stress granule assembly. J Virol 87: 1333–1347. doi: 10.1128/JVI.02263-12 23152511 10.1128/JVI.02263-12 PMC3554139

[33] Le Sage V, Cinti A, McCarthy S, Amorim R, Rao S, et al (2017) Ebola virus VP35 blocks stress granule assembly. Virology 502: 73–83. doi: 10.1016/j.virol.2016.12.012 28013103 10.1016/j.virol.2016.12.012

[34] Nelson EV, Schmidt KM, Deflube LR, Doganay S, Banadyga L, et al (2016) Ebola Virus Does Not Induce Stress Granule Formation during Infection and Sequesters Stress Granule Proteins within Viral Inclusions. J Virol 90: 7268–7284. doi: 10.1128/JVI.00459-16 27252530 10.1128/JVI.00459-16 PMC4984654

[35] Kedersha N, Cho MR, Li W, Yacono PW, Chen S, et al (2000) Dynamic shuttling of TIA-1 accompanies the recruitment of mRNA to mammalian stress granules. J Cell Biol 151: 1257–1268. 11121440 10.1083/jcb.151.6.1257 PMC2190599

[36] Dinh PX, Beura LK, Das PB, Panda D, Das A, et al (2013) Induction of stress granule-like structures in vesicular stomatitis virus-infected cells. J Virol 87: 372–383. doi: 10.1128/JVI.02305-12 23077311 10.1128/JVI.02305-12 PMC3536414

[37] Nikolic J, Civas A, Lama Z (2016) Rabies Virus Infection Induces the Formation of Stress Granules Closely Connected to the Viral Factories. 12: e1005942 doi: 10.1371/journal.ppat.1005942 27749929 10.1371/journal.ppat.1005942 PMC5066959

[38] Langereis MA, Feng Q, van Kuppeveld FJ (2013) MDA5 localizes to stress granules, but this localization is not required for the induction of type I interferon. J Virol 87: 6314–6325. doi: 10.1128/JVI.03213-12 23536668 10.1128/JVI.03213-12 PMC3648107

[39] McEwen E, Kedersha N, Song B, Scheuner D, Gilks N, et al (2005) Heme-regulated inhibitor kinase-mediated phosphorylation of eukaryotic translation initiation factor 2 inhibits translation, induces stress granule formation, and mediates survival upon arsenite exposure. J Biol Chem 280: 16925–16933. doi: 10.1074/jbc.M412882200 15684421 10.1074/jbc.M412882200

[40] Takahasi K, Yoneyama M, Nishihori T, Hirai R, Kumeta H, et al (2008) Nonself RNA-sensing mechanism of RIG-I helicase and activation of antiviral immune responses. Mol Cell 29: 428–440. doi: 10.1016/j.molcel.2007.11.028 18242112 10.1016/j.molcel.2007.11.028

[41] Ng CS, Jogi M, Yoo JS, Onomoto K, Koike S, et al (2013) Encephalomyocarditis virus disrupts stress granules, the critical platform for triggering antiviral innate immune responses. J Virol 87: 9511–9522. doi: 10.1128/JVI.03248-12 23785203 10.1128/JVI.03248-12 PMC3754122

[42] Onomoto K, Jogi M, Yoo JS, Narita R, Morimoto S, et al (2012) Critical role of an antiviral stress granule containing RIG-I and PKR in viral detection and innate immunity. PLoS One 7: e43031 doi: 10.1371/journal.pone.0043031 22912779 10.1371/journal.pone.0043031 PMC3418241

[43] Oh SW, Onomoto K, Wakimoto M, Onoguchi K, Ishidate F, et al (2016) Leader-Containing Uncapped Viral Transcript Activates RIG-I in Antiviral Stress Granules. PLoS Pathog 12: e1005444 doi: 10.1371/journal.ppat.1005444 26862753 10.1371/journal.ppat.1005444 PMC4749238

[44] Pham AM, Santa Maria FG, Lahiri T, Friedman E, Marie IJ, et al (2016) PKR Transduces MDA5-Dependent Signals for Type I IFN Induction. PLoS Pathog 12: e1005489 doi: 10.1371/journal.ppat.1005489 26939124 10.1371/journal.ppat.1005489 PMC4777437

[45] Zhang S, Jiang Y, Cheng Q, Zhong Y, Qin Y, et al (2017) Inclusion Body Fusion of Human Parainfluenza Virus Type 3 Regulated by Acetylated alpha-Tubulin Enhances Viral Replication. J Virol 91. 10.1128/JVI.01802-16 PMC5244348 27881643

[46] Zhang S, Sun Y, Chen H, Dai Y, Zhan Y, et al (2014) Activation of the PKR/eIF2alpha signaling cascade inhibits replication of Newcastle disease virus. Virol J 11: 62 doi: 10.1186/1743-422X-11-62 24684861 10.1186/1743-422X-11-62 PMC3994276

[47] Qin Q, Carroll K, Hastings C, Miller CL (2011) Mammalian orthoreovirus escape from host translational shutoff correlates with stress granule disruption and is independent of eIF2alpha phosphorylation and PKR. J Virol 85: 8798–8810. doi: 10.1128/JVI.01831-10 21715487 10.1128/JVI.01831-10 PMC3165827

[48] McInerney GM, Kedersha NL, Kaufman RJ, Anderson P, Liljestrom P (2005) Importance of eIF2alpha phosphorylation and stress granule assembly in alphavirus translation regulation. Mol Biol Cell 16: 3753–3763. doi: 10.1091/mbc.E05-02-0124 15930128 10.1091/mbc.E05-02-0124 PMC1182313

[49] Ariumi Y, Kuroki M, Kushima Y, Osugi K, Hijikata M, et al (2011) Hepatitis C virus hijacks P-body and stress granule components around lipid droplets. J Virol 85: 6882–6892. doi: 10.1128/JVI.02418-10 21543503 10.1128/JVI.02418-10 PMC3126564

[50] Khong A, Jan E (2011) Modulation of stress granules and P bodies during dicistrovirus infection. J Virol 85: 1439–1451. doi: 10.1128/JVI.02220-10 21106737 10.1128/JVI.02220-10 PMC3028890

[51] Montero H, Rojas M, Arias CF, Lopez S (2008) Rotavirus infection induces the phosphorylation of eIF2alpha but prevents the formation of stress granules. J Virol 82: 1496–1504. doi: 10.1128/JVI.01779-07 18032499 10.1128/JVI.01779-07 PMC2224440

[52] Borghese F, Michiels T (2011) The leader protein of cardioviruses inhibits stress granule assembly. J Virol 85: 9614–9622. doi: 10.1128/JVI.00480-11 21752908 10.1128/JVI.00480-11 PMC3165746

[53] Emara MM, Brinton MA (2007) Interaction of TIA-1/TIAR with West Nile and dengue virus products in infected cells interferes with stress granule formation and processing body assembly. Proc Natl Acad Sci U S A 104: 9041–9046. doi: 10.1073/pnas.0703348104 17502609 10.1073/pnas.0703348104 PMC1885624

[54] Abrahamyan LG, Chatel-Chaix L, Ajamian L, Milev MP, Monette A, et al (2010) Novel Staufen1 ribonucleoproteins prevent formation of stress granules but favour encapsidation of HIV-1 genomic RNA. J Cell Sci 123: 369–383. doi: 10.1242/jcs.055897 20053637 10.1242/jcs.055897

[55] Legros S, Boxus M, Gatot JS, Van Lint C, Kruys V, et al (2011) The HTLV-1 Tax protein inhibits formation of stress granules by interacting with histone deacetylase 6. Oncogene 30: 4050–4062. doi: 10.1038/onc.2011.120 21532619 10.1038/onc.2011.120

[56] Khaperskyy DA, Hatchette TF, McCormick C (2012) Influenza A virus inhibits cytoplasmic stress granule formation. Faseb j 26: 1629–1639. doi: 10.1096/fj.11-196915 22202676 10.1096/fj.11-196915

[57] Lindquist ME, Lifland AW, Utley TJ, Santangelo PJ, Crowe JE Jr. (2010) Respiratory syncytial virus induces host RNA stress granules to facilitate viral replication. J Virol 84: 12274–12284. doi: 10.1128/JVI.00260-10 20844027 10.1128/JVI.00260-10 PMC2976418

[58] Sun Y, Dong L, Yu S, Wang X, Zheng H, et al (2017) Newcastle disease virus induces stable formation of bona fide stress granules to facilitate viral replication through manipulating host protein translation. Faseb j 31: 1337–1353. doi: 10.1096/fj.201600980R 28011649 10.1096/fj.201600980R

[59] Heinrich BS, Cureton DK, Rahmeh AA, Whelan SP (2010) Protein expression redirects vesicular stomatitis virus RNA synthesis to cytoplasmic inclusions. PLoS Pathog 6: e1000958 doi: 10.1371/journal.ppat.1000958 20585632 10.1371/journal.ppat.1000958 PMC2891829

