# Supplementary figures and images for: Inclusion bodies of human parainfluenza virus type 3 inhibit antiviral stress granule formation by shielding viral RNAs
Source: PLoS Pathog. 2018 Mar 8;14(3):e1006948. doi: 10.1371/journal.ppat.1006948 (PMC5860793; doi:10.1371/journal.ppat.1006948)

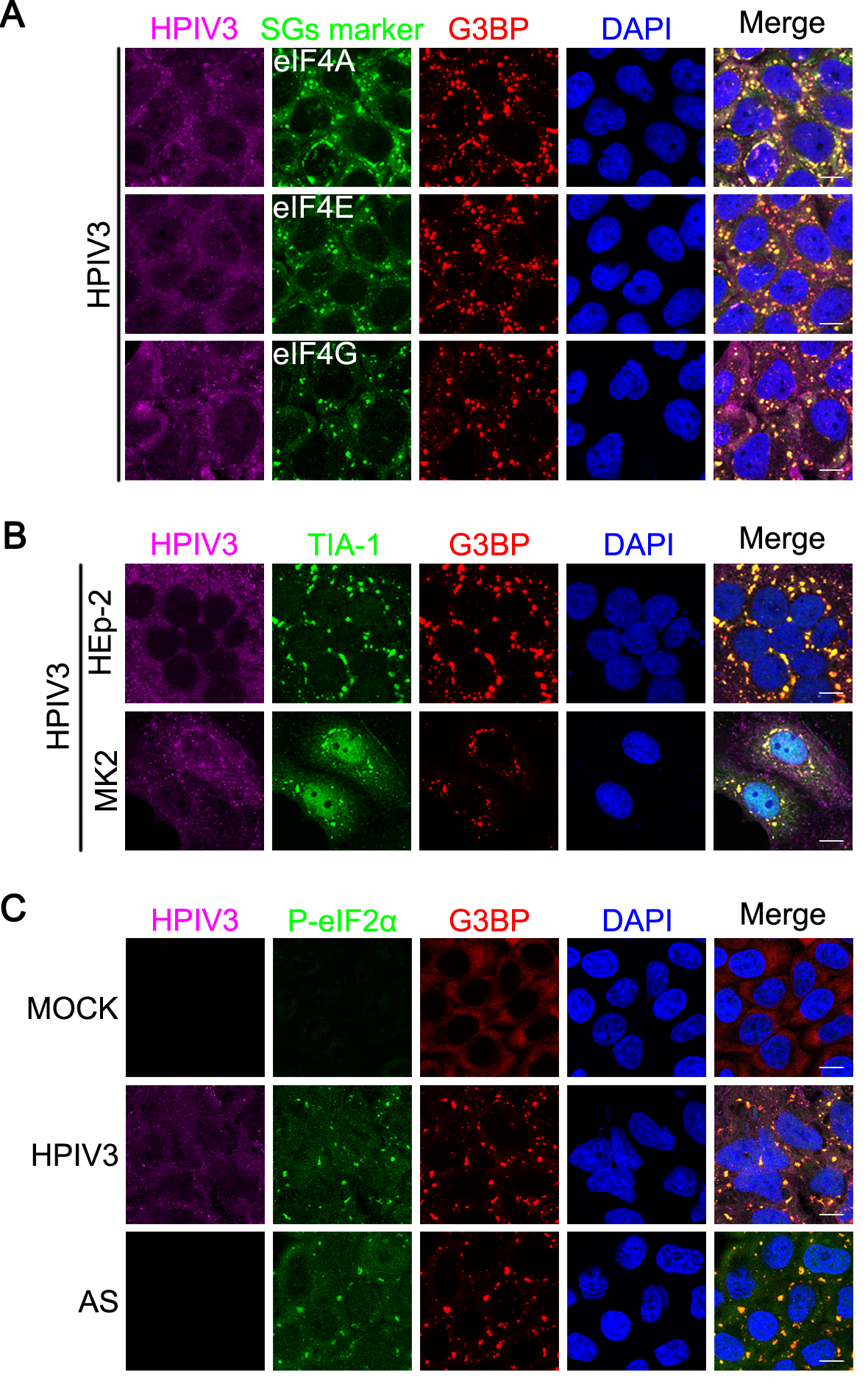

Supplement: S1 Fig — (A) HeLa cells were infected with HPIV3 (MOI = 1) for 24 h. Cells were immunostained for HPIV3 (purple), eIF4A/4E/4G (green), and G3BP (red). Nuclei were stained with DAPI (blue). The white scale bar corresponds to 10μm. (B) HEp-2 cells were infected with HPIV3 (MOI = 1), and MK2 cells were infected with HPIV3 (MOI = 0.1) for 24h. Cells were immunostained for HPIV3 (purple), TIA-1 (green), and G3BP (red). Nuclei were stained with DAPI (blue). The white scale bar corresponds to 10μm. (C) HeLa cells were mock-treated, infected with HPIV3 (MOI = 1) for 24h, or treated with AS (0.5 mM) for 1h. Cells were immunostained for HPIV3 (purple), phosphorylated eIF2α (green), and G3BP (red). Nuclei were stained with DAPI (blue). The white scale bar corresponds to 10μm. (TIF) [file ppat.1006948.s001.tif]

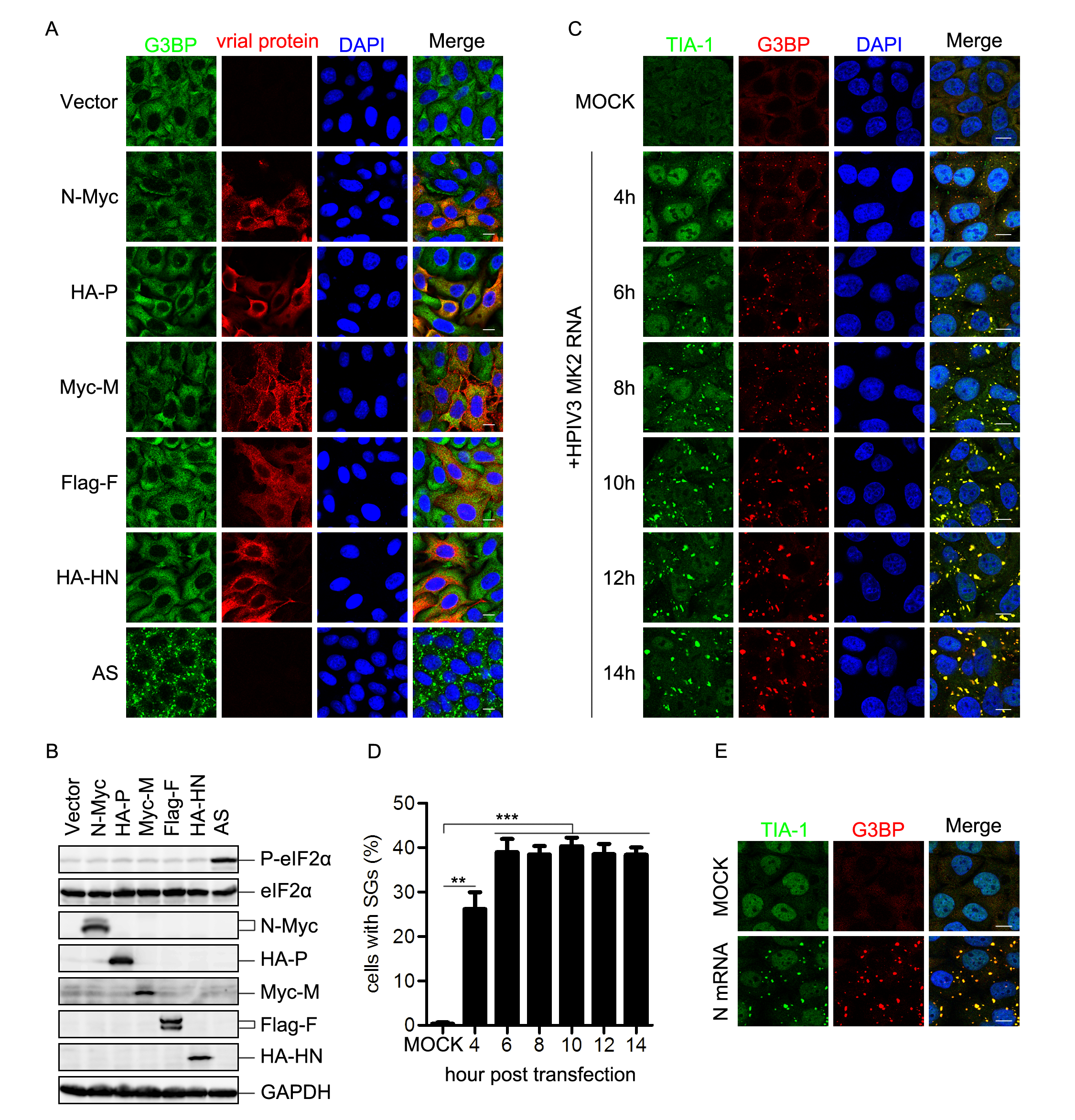

Supplement: S2 Fig — (A and B) HeLa cells were transfected with an empty plasmid or plasmids encoding N, P, M, F, or HN for 24 h or treated with AS for 1 h. (A) Cells were immunostained for G3BP (green) and Myc/HA/Flag tag (viral protein, red). Nuclei were stained with DAPI (blue). The white scale bar corresponds to 10μm. (B) Cell lysates were analyzed via western bot using anti-Myc, anti-HA, anti-Flag, anti-phosphorylated eIF2α, anti-eIF2α, and anti-GAPDH antibodies. (C and D) HeLa cells were transfected with the indicated RNA samples from HPIV3 infected MK2 cells. (C) Cells were immunostained for TIA-1 (green) and G3BP (red). Nuclei were stained with DAPI (blue). The white scale bar corresponds to 10μm. (D) The percentage of cells containing SGs was quantified in three independent experiments. (E) In vitro transcribed HPIV3 N mRNA was transfected into HeLa cells. Cells were immunostained for TIA-1 (green) and G3BP (red). Nuclei were stained with DAPI (blue). Data are represented as means ±SD. Student’s t test: * P<0.05, ** P<0.01, *** P<0.001, ns = not significant. (TIF) [file ppat.1006948.s002.tif]

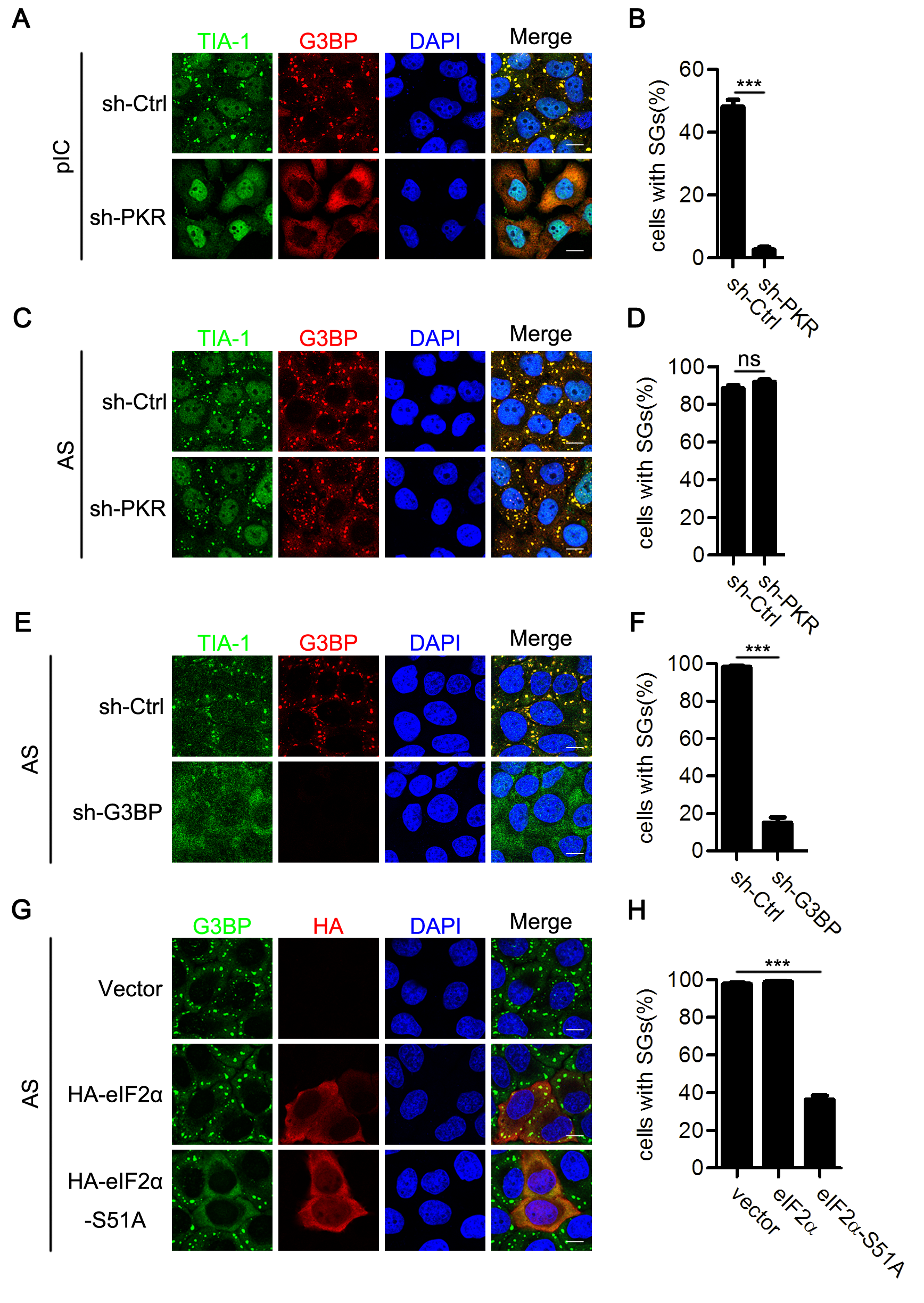

Supplement: S3 Fig — (A-D) HeLa cells with or without PKR knockdown were transfected with pIC for 12 h or treated with AS (0.5 mM) for 1 h. (A and C) Cells were immunostained for TIA-1 (green) and G3BP (red). Nuclei were stained with DAPI (blue). The white scale bar corresponds to 10μm. (B and D) The percentage of cells containing SGs was quantified in three independent experiments. (E and F) HeLa cells with or without G3BP knockdown were treated with AS (0.5 mM) for 1 h. (E) Cells were immunostained for TIA-1 (green) and G3BP (red). Nuclei were stained with DAPI (blue). The white scale bar corresponds to 10μm. (F) The percentage of cells containing SGs was quantified in three independent experiments. (G and H) HeLa cells were transfected with an empty plasmid or plasmids encoding eIF2α or the nonophosphorylatable mutant eIF2α-S51A for 24 h, then treated with AS (0.5 mM) for another 1 h. (G) Cells were immunostained for G3BP (green) and HA (red). Nuclei were stained with DAPI (blue). The white scale bar corresponds to 10μm. (H) The percentage of cells containing SGs was quantified in three independent experiments. Data are represented as means ±SD. Student’s t test: * P<0.05, ** P<0.01, *** P<0.001, ns = not significant. (TIF) [file ppat.1006948.s003.tif]

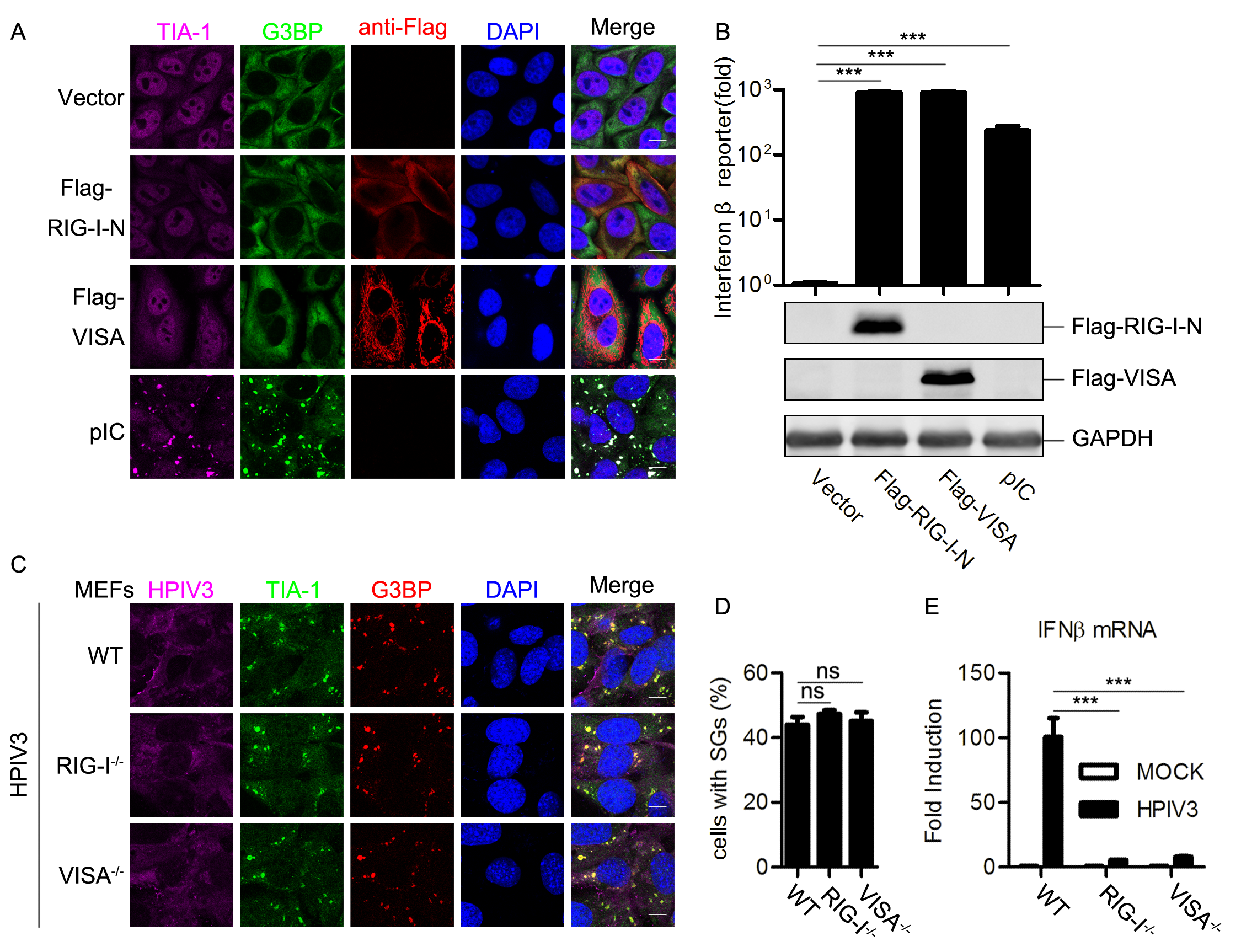

Supplement: S4 Fig — (A) HeLa cells were transfected with an empty plasmid or plasmids encoding RIG-I-N or VISA for 24 h or pIC for 12 h. Cells were immunostained for TIA-1 (purple), G3BP (green) and Flag (red). Nuclei were stained with DAPI (blue). The white scale bar corresponds to 10 μm. (B) HEK293T cells were transfected with 50 ng IFNβ-Luc reporter and 20 ng TK-Luc reporter together with the indicated plasmid encoding Flag-RIG-I-N or Flag-VISA or pIC for 24 h. Cells were harvested for a luciferase assay. Cell lysates were analyzed via western blot using anti-Flag and anti-GAPDH antibodies. (C-E) Wide type, RIG-I-/- or VISA-/- MEF cells were infected with HPIV3 (MOI = 1) for 24 h. (C) Cells were immunostained for HPIV3 (purple), TIA-1 (green) and G3BP (red). Nuclei were stained with DAPI (blue). The white scale bar corresponds to 10 μm. (D) The percentage of cells containing SGs was quantified in three independent experiments. (E) Total RNA were isolated for qPCR to determine the IFNβ mRNA abundance and normalized to that of GAPDH. Data are represented as means ±SD. Student’s t test: * P<0.05, ** P<0.01, *** P<0.001, ns = not significant. (TIF) [file ppat.1006948.s004.tif]

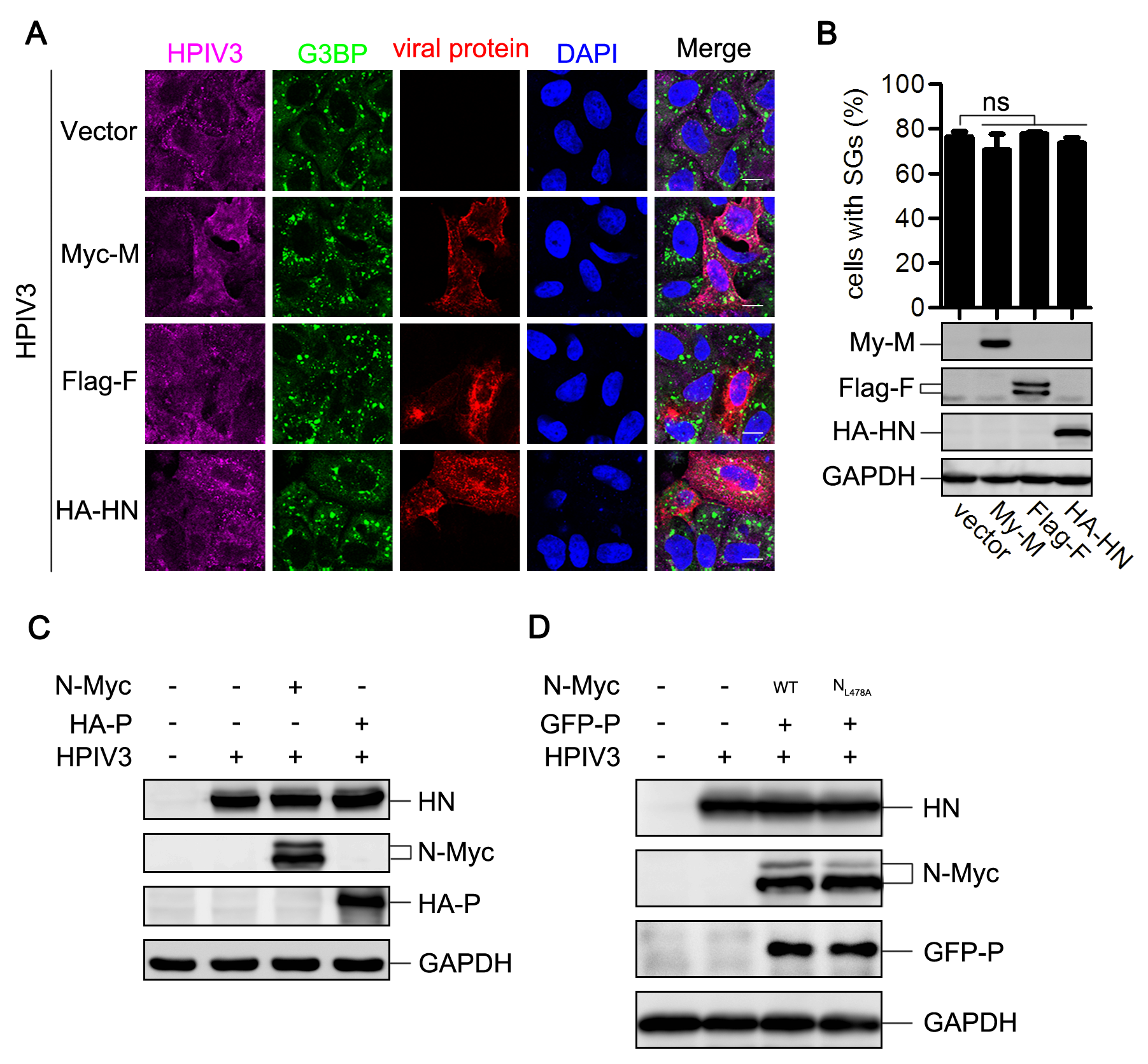

Supplement: S5 Fig — (A and B) HeLa cells were transfected with an empty plasmid or plasmids encoding M, F, or HN for 24 h, then infected with HPIV3 (MOI = 1) for another 24h. (A) Cells were immunostained for HPIV3 (purple), G3BP (green), and Myc/HA/Flag tag (viral protein, red). Nuclei were stained with DAPI (blue). The white scale bar corresponds to 10 μm. (B) The percentage of cells containing SGs was quantified in three independent experiments. Cell lysates were analyzed via western blot using anti-Myc, anti-Flag, anti-HA and anti-GAPDH antibodies. (C and D) HeLa cells were transfected with an empty plasmid or plasmids encoding N-Myc or HA-P or co-transfected with plasmids encoding GFP-P together with N-Myc or NL478A-Myc for 24 h, then mock infected or infected with HPIV3 (MOI = 1) for another 24 h. Cells lysates were analyzed using anti-HN, anti-Myc, anti-HA, anti-GFP and anti-GAPDH antibodies. Data are represented as means ±SD. Student’s t test: * P<0.05, ** P<0.01, *** P<0.001, ns = not significant. (TIF) [file ppat.1006948.s005.tif]

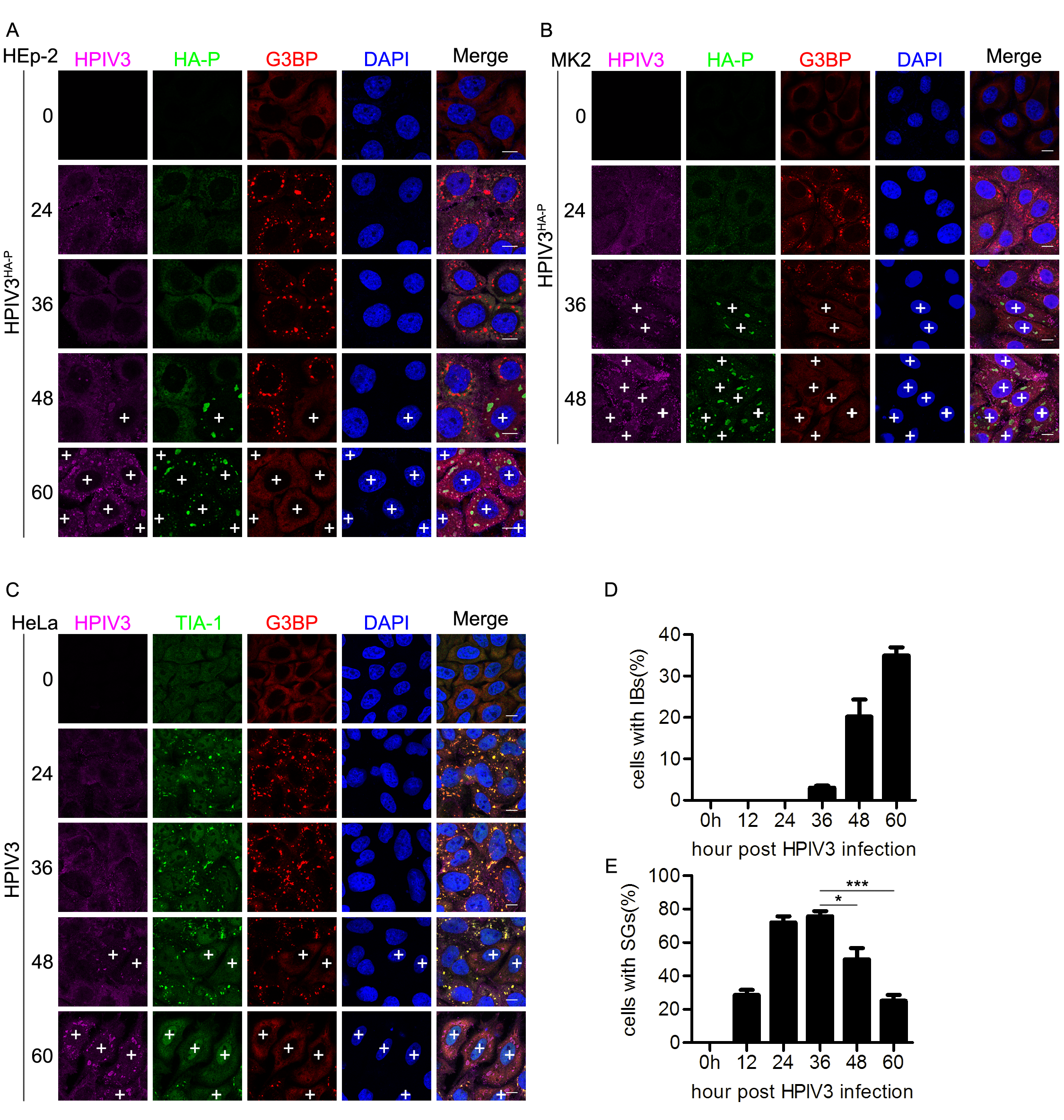

Supplement: S6 Fig — (A) HEp-2 cells were infected with HPIV3HA-P (MOI = 10), and (B) MK2 cells were infected with HPIV3HA-P (MOI = 1). At the indicated time points pi, cells were immunostained for HPIV3 (purple), HA (green), and G3BP (red). Nuclei were stained with DAPI (blue). The white scale bar corresponds to 10 μm. (C-E) HeLa cells were infected with HPIV3 (MOI = 1). (C) At the indicated time points pi, cells were immunostained for HPIV3 (purple), TIA-1 (green), and G3BP (red). Nuclei were stained with DAPI (blue). The white scale bar corresponds to 10 μm. (D and E) The percentage of cells containing IBs or SGs was quantified in three independent experiments. Data are represented as means ±SD. Student’s t test: * P<0.05, ** P<0.01, *** P<0.001, ns = not significant. (TIF) [file ppat.1006948.s006.tif]
